# Regulating Fe-spin state by atomically dispersed Mn-N in Fe-N-C catalysts with high oxygen reduction activity

**DOI:** 10.1038/s41467-021-21919-5

**Published:** 2021-03-19

**Authors:** Gege Yang, Jiawei Zhu, Pengfei Yuan, Yongfeng Hu, Gan Qu, Bang-An Lu, Xiaoyi Xue, Hengbo Yin, Wenzheng Cheng, Junqi Cheng, Wenjing Xu, Jin Li, Jinsong Hu, Shichun Mu, Jia-Nan Zhang

**Affiliations:** 1 College of Materials Science and Engineering, Zhengzhou University, Zhengzhou, 450001 PR China; 2 State Key Laboratory of Advanced Technology for Materials Synthesis and Processing, Wuhan University of Technology, Wuhan, 430070 PR China; 3 Foshan Xianhu Laboratory, Foshan, 528200 PR China; 4 International Joint Research Laboratory for Quantum Functional Materials of Henan Province, and School of Physics and Microelectronics, Zhengzhou University, Zhengzhou, 450001 PR China; 5 Canadian Light Source, 44 Innovation Blvd, Saskatoon, SK S7N 2V3 Canada; 6 Beijing National Laboratory for Molecular Sciences (BNLMS), CAS Key Laboratory of Molecular Nanostructure and Nanotechnology Institute of Chemistry, Chinese Academy of Sciences, Beijing, 100190 PR China

**Keywords:** Chemistry, Energy science and technology, Materials science, Nanoscience and technology

## Abstract

As low-cost electrocatalysts for oxygen reduction reaction applied to fuel cells and metal-air batteries, atomic-dispersed transition metal-nitrogen-carbon materials are emerging, but the genuine mechanism thereof is still arguable. Herein, by rational design and synthesis of dual-metal atomically dispersed Fe,Mn/N-C catalyst as model object, we unravel that the O_2_ reduction preferentially takes place on Fe^III^ in the FeN_4_ /C system with intermediate spin state which possesses one e_g_ electron (t_2g_4e_g_1) readily penetrating the antibonding π-orbital of oxygen. Both magnetic measurements and theoretical calculation reveal that the adjacent atomically dispersed Mn-N moieties can effectively activate the Fe^III^ sites by both spin-state transition and electronic modulation, rendering the excellent ORR performances of Fe,Mn/N-C in both alkaline and acidic media (halfwave positionals are 0.928 V in 0.1 M KOH, and 0.804 V in 0.1 M HClO_4_), and good durability, which outperforms and has almost the same activity of commercial Pt/C, respectively. In addition, it presents a superior power density of 160.8 mW cm^−2^ and long-term durability in reversible zinc–air batteries. The work brings new insight into the oxygen reduction reaction process on the metal-nitrogen-carbon active sites, undoubtedly leading the exploration towards high effective low-cost non-precious catalysts.

## Abstract (web summary)

The working mechanism of several low-cost electrocatalyst materials is still arguable. Here the authors show a model Fe,Mn/N-C catalyst where the oxygen reduction preferentially takes place on Fe(III) sites with the intermediate spin state (t2g4 eg1) caused by the adjacent Mn-N moieties.

## Introduction

The oxygen reduction reaction (ORR), represents the cornerstone for regenerative energy conversion devices involving polymer electrolyte membrane fuel cell (PEMFC) and metal–air batteries^1–7^. Hitherto, the generally recognized state-of-the-art platinum (Pt)-based catalysts possess the highest kinetic activity in catalyzing ORR under acid and alkaline media, however, the scarcity, price, and low methanol crossover tolerance of Pt alloys have motivated the search for cost-effective non-noble-metal electrocatalysts^8–13^. Replacement of noble-metal materials with less expensive, highly active, and durable electrocatalysts for ORR is thereby increasingly attractive but arduous with great challenges ahead^14–17^.

Transition metals (M = Mn, Fe, Co, Ni, etc.) possessing the 3*d* unoccupied orbitals can accommodate foreign electrons to reduce the bonding strength between OOH*,O*/OH* intermediates, allowing them with the potential to catalyze the O_2_ reduction process^18^. During such a process, the activity of catalysts is mainly affected by its electronic structure, where the energy is released to form the M^(m+1)+^-O_2_^2−^ bond on the surface of catalysts by breaking the M^m+^-OH^−^ bond, ensuring a fast displacement of O^2−^/OH^−^ and OH^−^ regeneration^19,20^. Generally, Fe^III^ possesses multiple states due to the coordination environment, which can be classified to display different forms of spin (low spin t_2g_5 e_g_0, medium spin t_2g_4 e_g_1, and high spin t_2g_3 e_g_2)^21^. The low-spin electron configuration is d_xy_2 d_yz_2 d_xz_1, without electrons occupying anti-bond orbitals, resulting in strong M^m+^/O_2_ interactions and stable M^(m+1)+^-O_2_^2−^ bonds, and making it difficult for the M^(m+1)+^-O_2_^2−^/M^m+^-OOH transition^22^. The electron configuration of high spin is d_xy_1 d_yz_1 d_xz_1 d_z_^2^1 d_x_^2^-_y_^2^1. Unfortunately, the high eg filling (d_z_^2^1 d_x_^2^-_y_^2^1) results in poor adsorption ability and bad performance. The medium spin electron configuration is d_xy_2 d_yz_1 d_xz_1 d_z_^2^1, while the single d_z_^2^ electron of the mediate spin state can readily penetrate the antibonding π-orbital of oxygen, rendering high ORR activity^23^. To date, a large amount of research has been devoted to the identification and geometric design of active sites to expose more active sites^24^. However, it is rarely reported to improve the activity of the catalyst by regulating the electronic structure. Therefore, modulating metal species with moderate spins is expected to increase ORR activity, but how to easily control the spin state is still very challenging^25^.

The family of metal-nitrogen complex carbon (M–N–C) materials has high conductivity and unique metal–ligand interaction, which have been regarded as the most promising alternatives to commercial Pt/C because of their outstanding performance in activation of oxygen^26–30^. According to the latest research, due to the improvement in the structural stability of the active center and the modulation of the electron cloud, bimetallic particles show higher activity and stability when compared with monometallic atomic particles^31–33^.

**Fig. 1:**
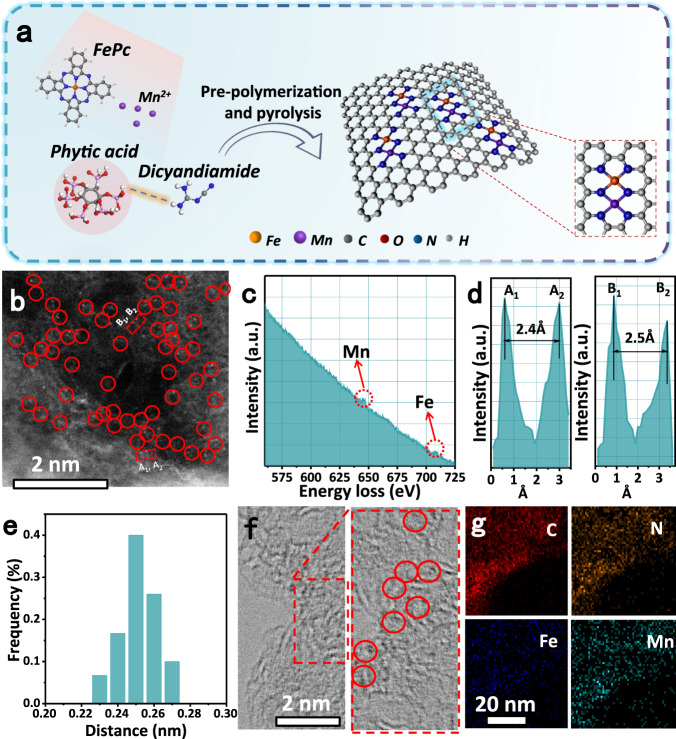
Synthetic illustration and TEM characterizations of Fe,Mn/N–C catalyst. **a** Schematic illustration of synthesis procedure for Fe,Mn/N–C catalysts. **b** Aberration-corrected HAADF-STEM image and some of bimetallic Fe/Mn sites are highlighted by larger red circles. **c** Fe,Mn/N–C structure analyzed by EELS. **d** The intensity profiles obtained on two bimetallic Fe–Mn sites. **e** Statistical Fe–Mn distance in the observed diatomic pairs. **f** HR-TEM of Fe,Mn/N–C, in which some lattice distortions are highlighted by red circles. **g** HAADF-STEM image of Mn, Fe/N–C with mappings of individual elements (C, N, Fe, and Mn).

Herein, we first successfully implant Mn–N moieties in the conventional Fe/N–C system, by preparing a dual-metal atomically dispersed Fe,Mn/N–C electrocatalyst with the pre-polymerization and pyrolysis processes, whereby dicyandiamide is used as C and N sources, iron phthalocyanine (FePc) and manganese nitrate (Mn(NO_3_)_2_) are selected as metal precursor. The dual-sites dispersion tagged in the N-doped defect carbon is probably originated from adsorption of Mn salts and their bonding with neighboring Fe–N_4_ center (Iron phthalocyanine molecular) (Fig. 1a)^15^. The introduction of Mn–N moieties causes Fe^III^ electron delocalization and makes the spin state of Fe^III^ transition from low spin (t_2g_5 e_g_0) to intermediate spin (t_2g_4 e_g_1), readily penetrating the antibonding π-orbital of oxygen, and thus allowing an excellent ORR activity in both 0.1 M HClO_4_ and 0.1 M KOH solutions. DFT calculations reveal that Fe,Mn/N–C can interact with oxygen moderately, with appropriate bond length and adsorption energy, beneficial to promote the kinetic process of ORR.

## Results

### Material synthesis and characterization

The single metal atomically dispersed Fe/N–C and Mn/N–C catalysts with the sample loading using the same method were also synthesized as controlled experiments. As shown in Supplementary Fig. 1, the scanning electron microscopy (SEM) and transmission electron microscopy (TEM) images of Fe,Mn/N–C show a graphene-like carbon sheet morphology with a porous structure^34^. The aberration-corrected high-angle annular dark-filed scanning TEM (HAADF–STEM) was used to acquire the evidence of Fe and Mn distribution at atomic resolution. As displayed in Fig. 1b, a number of bright spots are clearly observed, in which Fe/Mn atomic pairs are randomly distributed on the surface of N-doped carbon as highlighted by red cycles, attributed to heavy Fe, Mn than light N, C atoms^35^. In order to make it clearer whether Mn–N_4_ is adjacent to Fe–N_4_, the atomic-resolution HAADF-STEM of Fe–N–C and Mn–N–C catalysts have been additionally carried out. As displayed in Supplementary Figs. 2 and 3, isolated single Fe or Mn atoms can be distinctly observed on the carbon support, indicating that neither atomic Fe–Fe pairs nor atomic Mn–Mn pairs form by our synthetic strategy. In contrast, when adding Mn and Fe sources stimulatingly in the synthetic system, a number of bright dual spot-pairs are clearly observed, further confirming that it forms the heterogeneous-metal- atoms pairs (Supplementary Fig. 4). The electron energy loss spectrum (EELS) (Fig. 1c) reals the coexistence of Fe and Mn elements in Fe,Mn/N–C. As shown in Fig. 1d–e, a statistical analysis of more than 30 metal pairs has been conducted, which presents that the distance between the metal-pairs is 0.25 ± 0.02 nm. The high-resolution TEM (HRTEM) images (Fig. 1f) demonstrates the lattice distortion defect characteristic, which might be attributed to the coordination of dual Fe/Mn atoms with nitrogen. The energy-dispersive X-ray spectroscopic (EDS) elemental mapping analyses further manifest the homogeneous distribution of C, N, Fe, and Mn distribution over Fe,Mn/N–C (Fig. 1g). No characteristic crystal peaks of metal and metal oxides can be observed in the X-ray reduction patterns of carbonized samples, excluding the formation of large particles (Supplementary Fig. 6)^36^. Taken together, such collective HAADF-STEM, EDS elemental mapping, EELS and the distance between the metal-pairs confirm the co-existence of Fe and Mn in a form of Fe/Mn atomic pairs^37^.

The Raman spectrum of Fe,Mn/N–C shows that the intensity ratio of two main bands located at 1354 and 1591 cm^−1^ (*I*_D_/*I*_G_) is 0.94, further confirming the defective structure of the carbon nanosheets^38^ (Supplementary Fig. 7, Supplementary Table 1). The specific surface area and pore volume of Fe,Mn/N–C, with rich microporous and mesoporous, are 245.33 m^2^g^−1^ and 0.3 m^3^g^−1^, respectively (Supplementary Fig. 8). As reported, microporous can increase the density of active sites, and mesoporous are beneficial to the mass transfer, thus improving catalytic activity^39^.

The chemical composition of Fe,Mn/N–C was further investigated using X-ray photoelectron spectroscopy (XPS). As depicted in Supplementary Table 3, after rational doping of Mn^III^ moieties, the content of graphitic N (401.63 eV) increases compared with Fe–N/C sample, implying that Mn tends to catalyze the formation of highly ordered and less defective graphitic carbon, thereby improving the stability of nanocarbons^40^. As depicted in Supplementary Fig. 9a, the N 1s spectrum indicates five types of N species existing in Fe,Mn/N–C. Especially, the Fe/Mn–N_x_ bond with the binding energy of 399.1 eV, associated with the abundant atomically dispersed metal-nitrogen functional moieties in Fe,Mn/N–C. The content of Fe and Mn detected by ICP analysis are 2.3 and 1.6 wt. %, respectively.

**Fig. 2:**
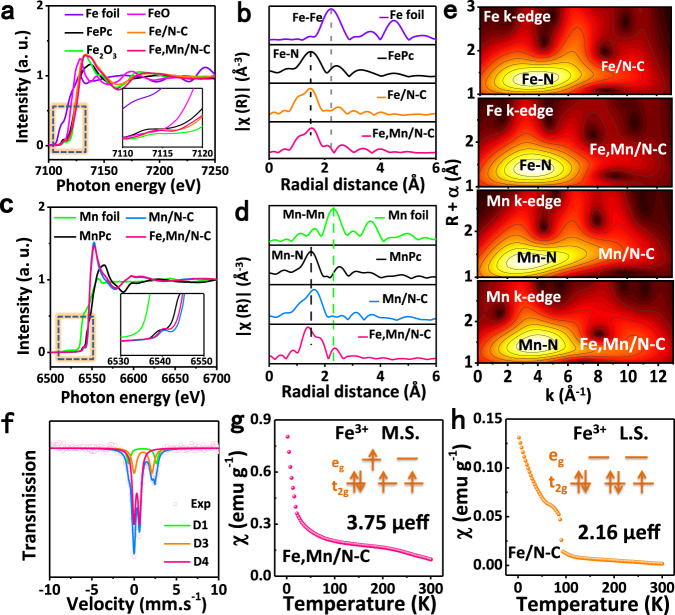
XAS and 57Fe Mössbauer spectroscopy and Magnetic susceptibility of the catalysts. **a** Fe K-edge XANES and **b** Fourier-transform EXAFS spectra of Fe,Mn/N–C and reference samples. **c** Mn K-edge XANES and **d** Fourier-transform EXAFS spectra of Fe,Mn/N–C and reference samples. **e** Wavelet transform of the k^3^-weighted EXAFS data of Fe,Mn/N–C, Fe/N–C, and Mn/N–C. **f** Room-temperature ^57^Fe Mossbauer spectrum of Fe,Mn/N–C. Magnetic susceptibility of **g** Fe,Mn/N–C, **h** Fe/N–C (M.S. represents medium-spin, L.S. represents low-spin).

To make it clear of the local structural information for Fe and Mn, X-ray absorption near-edge structure (XANES) and K-edge extended X-ray absorption fine structure (EXAFS) measurements were performed^41^. As shown in Fig. 2a, Fe K-edge XANES spectra show that the adsorption threshold position of Fe,Mn/N–C and Fe/N–C located between FeO and Fe_2_O_3_, which indicated that the valance of Fe in Fe,Mn/N–C and Fe/N–C is situated between +2 and +3^42^. As shown in Fig. 2b, the Fourier transform (FT) k^3^-weighted EXAFS spectra of Fe K-edge show apparent the same peak of Fe,Mn/N–C and Fe/N–C at 1.4–Å corresponding to the Fe–N coordination. The peak at 2.16–Å found in Fe,Mn/N–C represents the average nearest Fe-metal atoms distance but almost no such signal in Fe/N–C at the same position. As shown in Fig. 2d, the Fourier transform (FT) k^3^-weighted EXAFS spectra of Mn K-edge demonstrate that Fe,Mn/N–C not only shows a peak at 1.40 Å indexing Mn–N coordination, but also gives a peak at 2.34 Å, demonstrating the next nearest Mn–metal atoms distance in Fe,Mn/N–C. Wavelet transform (WT) was also used to investigate the Fe K-edge EXAFS oscillations of Fe,Mn/N–C and the references. As shown in Fig. 2e, the WT analysis of Fe,Mn/N–C, Fe/N–C and Mn/N–C shows only one intensity maximum at about 4.0 Å^–1^ which is very close to that in the reference FePc (~4.0 Å^–1^) and MnPc (~4.0 Å^–1^) (Supplementary Fig. 11a, c). In order to confirm the existence of Fe,Mn–N_6_-1 moieties in Fe,Mn/N–C, density functional theory (DFT) is firstly used to deduce the possible structure of Fe,Mn/N–C (Supplementary Fig. 12a–e) and single atom Fe–N_4_ (Supplementary Fig. 12f). Based on its simulated Fe K-edge spectra of XANES, the calculated architectural Fe,Mn–N_6_-1 model was in agreement with experimental spectra. Other structures could be excluded by the comparisons between the K-edge XANES experimental and theoretical spectra (Supplementary Fig. 12). Therefore, the Fe,Mn/N_6_-1 model is suggested as the most possible structure for our catalyst. Furthermore, as calculated based on the fitting k space curve in Supplementary Fig. 13 and listed in Supplementary Table 4, the coordination numbers of Fe–N_1_, Fe–N_2_ and Fe–Mn are 1.8 ± 0.3, 2.0 ± 0.4, and 0.9 ± 0.2, respectively, and the coordination numbers of Mn–N_1_, Mn–N_2_, and Mn–Fe are 1.9 ± 0.3, 2.2 ± 0.4, and 1.1 ± 0.2, respectively. Compared with reported single metal centers in M–N–C structures, Fe,Mn/N–C adopted a different dual-metal center, whereas the porphyrin-like structure was deformed. Thus, it is reasonable that the bond distances of Fe–N_1_, Fe–N_2_, Mn–N_1_, and Mn–N_2_ are different. Consequently, it can be inferred that the structure of Fe,Mn/N–C is Fe,Mn/N_6_-1 model.

To discriminate different Fe species, Mossbauer spectroscopy analysis was carried out^43^. As shown in Fig. 2f, for the Mossbauer spectra of Fe,Mn/N–C, D_1_ with relatively larger values of isomer shift (IS) and quadrupole splitting (QS), can be assigned to FePc-like Fe^II^ N_4_ species^44^. D_3_ can be attributed to the N-(Fe^Ш^N_4_)-N high-spin structure, the structure is so robust that it cannot be acted as the catalytically active site. D_4_ can be assigned to the N-(Fe^Ш^N_4_) medium-spin structure, and the unsaturated coordination structure of D_4_ enables it to be catalytically active in chemical reactions. The IS(δ), descriptor of the valence state of the Mossbauer absorber atom, indicates that Fe exists mainly in the form of trivalent with high crystal field stabilization energy^25^. The quantitative analysis (Supplementary Table 5) reveals that the iron responsible for D_1_, D_3,_ and D_4_ makes up 13.5%, 27.1%, and 59.4%, respectively. This confirms that the Fe^III^ in Fe,Mn/N–C is predominantly present as Fe^III^ with the medium-spin structure.

To further unravel the electron spin configuration of Fe,Mn/N–C, the zero-field cooling (ZFC) temperature-dependent magnetic susceptibility was measured^24^. The calculated effective magnetic moment of Fe,Mn/N–C and Fe/N–C is 3.75 *μ*_eff_ and 2.16 *μ*_eff_, respectively (Fig. 2g, h). Besides, we further obtained the number of unpaired *d* electron (*n*) of Fe^III^ ion via the following equation:^22^

1 $$\mu _{{\mathrm{eff}}} = \sqrt {n(n + 2)} $$

whereby the number of unpaired d electron (*n*) of Fe/N–C is about 1.3, which means Fe^III^ ions have a low-spin state without e_g_ filling, so that no electron occupied in the σ* antibonding orbital of FeN_4_ leads to a very strong Fe^III^/O_2_ interaction and a quite stable Fe^4+^-O_2_^2−^ bond^18^. While the number of unpaired *d* electron (*n*) of Fe,Mn/N–C is about 3, which has single e_g_ filling^21^. Certainly, the unusual low-spin state of neighboring Mn^III^ moieties permits Fe^III^ in FeN_4_ to achieve the ideal e_g_ filling. It is reckoned that this intrinsically optimal electronic configuration would endow the as-designed Fe,Mn/N–C with a high catalytic activity. For verification, we measure the temperature-programmed desorption of O_2_ (O_2_-TPD) as shown in Supplementary Fig. 14. Owing to the electron affinity of oxygen, the electron can be transferred from catalyst to chemisorbed oxygen, so that it requires high temperature for desorption^24^. The amount and the O_2_ desorption temperature obviously increase from Fe,Mn/N–C (O_2_ desorption temperature is 385.4 °C) to Fe/N–C (O_2_ desorption temperature is 407.6 °C), indicative of stronger bonding between the O_2_ and the Fe/N–C than that of Fe,Mn/N–C, which is consistent with the ZFC results.

To probe the interaction between anchored Fe and Mn, the density of states (DOS) near the Fermi level, mainly originated from the 3*d* state, was investigated^45^. As shown in Supplementary Fig. 15, obviously, sharp peaks can be seen near the Fermi level for both Fe (Fe–N_4_) and Mn (Mn–N_4_), and the peaks for Fe are more sharply. These indicated that the interaction between Fe and the coming O_2_ are stronger than that on Mn. For Fe,Mn/N_6_-1, clearly differences can be seen, there are obvious overlapping between Fe 3*d* and Mn 3*d*, which reflected the interaction by the neighboring atom. In addition, it is supposed that the antibonding state of Fe/N–C possesses low electron energy giving low-spin state, while the higher antibonding electron energy of Fe,Mn/N–C further confirming the medium-spin configuration of Fe^III^ in Fe,Mn/N–C. This probably owing to that Mn^III^ with higher affinity to electrons can capture electrons from Fe^III^, thus leading to redistribution electrons of Fe 3*d*^46^. In addition, the orbital hybridization of Fe 3*d* and Mn 3*d* causes obvious band gap narrowing and electron delocalization in Fe,Mn/N–C, which increases the conduction band dispersion and reduces the large effective electron mass, thereby improving the electron transport.

### Electrochemical oxygen reduction performance

**Fig. 3:**
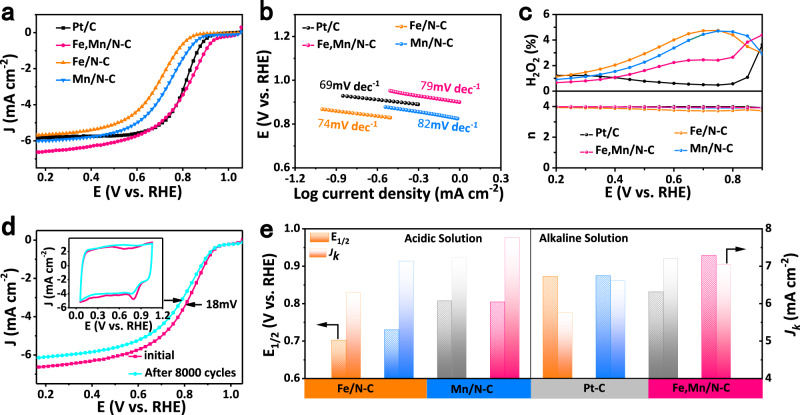
ORR performances of Fe,Mn/N–C in 0.1 M HClO4 and 0.1 M KOH. **a** LSV curves of Fe,Mn/N–C, Fe/N–C, Mn/N–C and Pt/C catalyst in O_2_-saturated 0.1 M HClO_4_ solution. **b** Corresponding Tafel plots obtained from the RDE polarization curves. **c** H_2_O_2_ yield and electron transfer number (*n*) in 0.1 M HClO_4_ solution. **d** ORR polarization LSV and CV curves of Fe,Mn/N–C measurement before and after 8000 potential cycles at the scan rate of 50 mV s^−1^ with the rotation speed of 1600 rpm. **e** Comparison of the kinetic current density (*J*_k_) and E_1/2_ of Fe,Mn/N–C, Fe/N–C, Mn/N–C, and Pt/C catalysts.

To verify the oxygen reactivity of Fe,Mn/N–C with the medium-spin structure for Fe^III^, the ORR electrocatalytic activity in 0.1 HClO_4_ aqueous solution of Fe,Mn/N–C was first demonstrated by compared with Fe/N–C and Mn/N–C with Cyclic voltammetry (CV) and Linear scan voltammetry (LSV) measurements^47^. CV curve reveals a significant reduction peak at 0.804 V for Fe,Mn/N–C (Supplementary Fig. 16), suggesting a good ORR electrocatalytic activity. LSV curves in O_2_-saturated 0.1 M HClO_4_ with a loading amount of 0.1 mg cm^−2^ was performed (Fig. 3a). Fe,Mn/N–C obviously presents a high half-wave potential (E_1/2_) of 0.804 V (Supplementary Table 6), which are similar to Pt/C (E_1/2_ = 0.807 V), and superior to Fe/N–C (E_1/2_ = 0.702 V), Mn/N–C (E_1/2_ = 0.73 V) and most of non-precious metal ORR electrocatalysts (Supplementary Table 7). The ORR kinetics was further probed by the Koutecky–Levich (K–L) method (Supplementary Fig. 17) and Tafel plot (Fig. 3b). The electron transfer number (n) of Fe,Mn/N–C is 3.91, as evaluated according to the K-L equation^12^. The n and H_2_O_2_ yield were further evaluated through rotating ring-disk electrode (RRDE, Fig. 3c), where the direct four-electron transfer mediated ORR process is confirmed (the n approaches 4 and the H_2_O_2_ yield is below 4%).

**Fig. 4:**
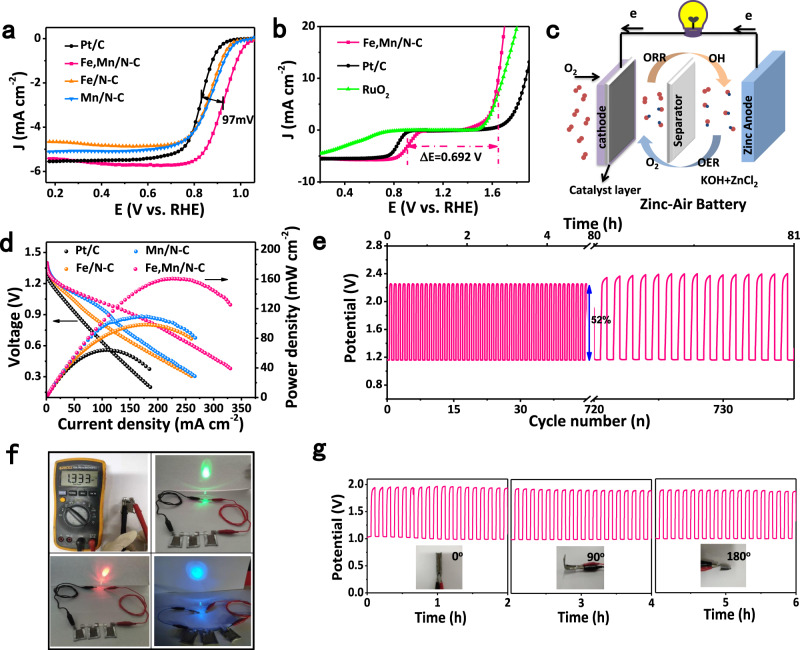
The performance of Zn–Air Batteries system of Fe,Mn/N–C catalyst. **a** LSV curves of Fe,Mn/N–C, Fe/N–C, Mn/N–C, and Pt/C catalyst in O_2_-saturated 0.1 M KOH solution. **b** LSV curves of Fe,Mn/N–C, commercial Pt/C, and RuO_2_ catalysts on an RDE in 0.10 M KOH, indicating the bifunctional activities toward both ORR and OER. **c** Schematic representation of the liquid rechargeable ZAB. **d** Polarization and power density curves of the primary Zn–air batteries of the Fe,Mn/N–C, Fe/N–C, Mn/N–C and Pt/C catalyst in O_2_-saturated 6 M KOH solution. **e** Charge−discharge cycling performance of rechargeable Zn−air batteries at a constant charge−discharge current density of 5 mA cm^−2^. **f** Photograph of all-solid-state zinc–air battery displaying a measured open-circuit voltage of ~1.333 V. Photograph of a lighted LED powered (Left to right be green, red and blue) by three all-solid-state Zn–air batteries. **g** Galvanostatic discharge–charge cycling curve at 1 mA cm^−2^ for the all-solid-state rechargeable ZAB, applying bending strain (as depicted by the inset images) every 2 h.

Also, Fe,Mn/N–C exhibits excellent stability, as evidenced by a loss of only 18 mV in E_1/2_ after 8000 potential cycles in O_2_-saturated 0.1 M HClO_4_ solution (Fig. 3d). The stability is largely enhanced over Fe/N–C (loss 34 mV after 8000 cycles), as well as the Mn/N–C (21 mV loss) (Supplementary Fig. 18). It can be attributed to the promotion of Mn to the formation of graphitized carbon and MnO_2_ during the OER process, enhancing the stability of nano-carbon with reduced carbon corrosion^48^. In addition, Fe,Mn/N–C displays a benchmark chronoamperometry response extended over 40,000 s with retention of 96% of the initial current (Supplementary Fig. 19). Interestingly, Fe,Mn/N–C exhibits more stable retention of current densities without a distinct recession after methanol crossover and CO, implying strong resistance to corrosion and poisoning in acidic electrolytes (Supplementary Fig. 20), better than Pt/C (20 wt%) catalyst and control samples (Fe/N–C and Mn/N–C)^49^. Significantly, Fe,Mn/N–C also presents outstanding ORR activity measured by CV, LSV, and Tafel plot in 0.1 M KOH (Supplementary Fig. 21–23a). It shows high ORR catalytic activity with E_1/2_ of 0.928 V (Fig. 4a, Supplementary Table 8 and 9), 97 mV superior to that of Pt/C (E_1/2_ = 0.831 V). The H_2_O_2_ yield for the Fe,Mn/N–C remains below 3% over all potentials with *n* ≈ 4, comparable to Pt/C (Supplementary Fig. 23b)^49^. What should be noted that Fe,Mn/N–C exhibits excellent with almost no activity decay after 40,000 CV scanning cycles in O_2_-saturated 0.1 M KOH solution (Supplementary Fig. 23c). In addition, Fe,Mn/N–C also exhibiting the strong tolerance to methanol with long-term current stability (Supplementary Fig. 23d, e)^50^.

### The Zn–Air Batteries system analysis

The overall oxygen electrode activity can be evaluated by the difference of OER and ORR metrics^51^.

2 $$\Delta E = E_{{\mathrm{j}} = 10} - E_{1/2}$$

Fe,Mn/N–C exhibits a ΔE value of 0.692 V, lower than RuO_2_ and Pt/C (Fig. 4b). Inspired by the excellent OER and ORR performance, we assembled liquid and flexible all-solid-state rechargeable ZABs for practical applications of Fe,Mn/N–C (Fig. 4c)^52^. As anticipated, it displays a high open-circuit voltage of up to 1.4 V and a peak-power density as high as 160.8 mW cm^−2^ (Fig. 4d), superior to commercial Pt/C (≈64 mW cm^−2^)^53^. Surprisingly, Fe,Mn/N–C has a specific capacity of 902 mAh g zn^−1^ at the discharge current density of 5 mA cm^−2^ with a corresponding energy density of 1136.5 Wh kgzn^−1^ (Supplementary Fig. 25). Its excellent stability is further illustrated by over 81 h at 5.0 mA cm^−2^ (Fig. 4e), where the voltage barely changes. A high OVC of 1.33 V is obtained in Fig. 4f. The all-solid-state battery exhibited stable charge (1.94 V) and discharge (1.04 V) potentials at the current density of 1 mA cm^−2^ for 6 h even when the device was bended to a large angle or folded back to front (Fig. 4g)^54^.

### Atomistic insight into the Fe,Mn/N–C activities

**Fig. 5:**
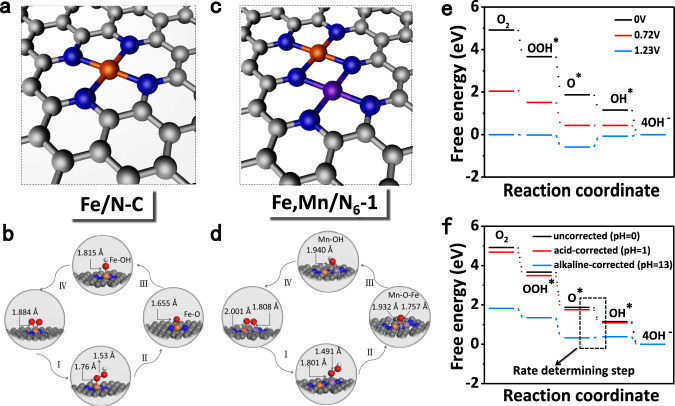
DFT calculations of the ORR activity on Fe,Mn/N–C and Fe/N–C catalysts. The optimized structure of (**a**) Fe/N–C and (**c**) Fe,Mn/N–C. Optimized atomic structures for the main process of an ORR: **b** Fe/N–C and **d** Fe,Mn/N–C. **e** The pathways for Fe,Mn/N–C are summarized at U = 0 V, 0.72 V, and 1.23 V, respectively. **f** pH-corrected free energy diagram of the Fe,Mn/N_6_-1.

To further shed light on the reason of the superior ORR activities of Fe,Mn/N–C, density functional theory (DFT) calculations were conducted. Supplementary Figs. 26–28 and Supplementary Tables 10–12 show all possible active sites by optimized the structure via DFT calculations and their free energies for each elementary step was calculated by combining the enthalpy and the harmonic entropy. Considering Fe,Mn/N_6_-1, is the nearest structure of our dual-metal atomically catalyst, we apply Fe,Mn/N_6_-1, FeN_4_, and MnN_4_ graphene as the model reference to represent the difference of Fe,Mn/N–C, Fe/N–C, and Mn/N–C (Supplementary Figs. 27 and 28). In the first step, the metal active site adsorbs oxygen molecules and forms a long Mn–O_2_ bond (2.209 Å) in Mn/N–C (Supplementary Fig. 30). Due to the large Mn^III^ ion radius, the interaction with oxygen is so weak that the kinetic rate is limited because the process of proton-electron transfers to *O_2_ or splitting of the *O bind in O_2_ (dissociative mechanism) demand extra energy. In addition, Mn^III^ ions are unstable in solution and underwent a disproportionation reaction to form Mn^II^ and Mn^IV^ ions, which makes it very low ORR activities and low stability. The Fe–O_2_ bond is 1.884 Å in Fe/N–C (Fig. 5a, b), which makes reaction site that bind oxygen too strongly and proton-electron transfer *O or *OH be circumscribed^42^. This promotes the formation of more peroxide intermediates and leads to poor ORR activity and stability. While oxygen is adsorbed by Fe/Mn atom pair (Fig. 5c, d), it leads to proper bond length and suitable binding energy, which would reduce the dissociation energy barrier. In addition, it can effectively capture oxygen-containing intermediates and quickly break the bond between M–OH to ensure the regeneration of O*, OH*, making the ORR kinetics faster by effectively inhibiting the production of peroxides^40^. The calculated minimum free-energy path along the subtractions of the ORR is listed in Supplementary Fig. 26. For the Fe,Mn/N_6_-1 model at a potential U = 0 V, all the reaction steps from O_2_ to OH^−^ are downhill, implying a facile reaction. At U = 0.72 V, the subtraction step from O* to OH* takes place, while the other subtractions remain downhill. This potential is consequently called the thermodynamic limiting potential. The limiting potentials are determined to be 0.42 V and 0.36 V for Mn/N–C and Fe/N–C, respectively. Therefore, it suggests that Fe,Mn/N–C with a highly reactive ORR process under this condition, consistent with our experimental results. In addition, as shown in Fig. 5e, f at a pH different from 0, the acid-corrected one does not significantly change the free energy diagram, while the alkaline-corrected one does. However, the pH-corrected operation would not change the rate-determining step of oxygen reduction reaction with the same calculation model. Accordingly, despite the numerical change of overpotential, the theoretical ORR trend would not change for different models as well, as a result of the same pH-corrected value. That is to say, the effect of electrolyte would not change the current conclusion: the Fe,Mn/N_6_-1 model possess the best theoretical ORR activity with the smallest overpotential among all models. In addition, Supplementary Fig. 31 and Supplementary Table 15 show all possible active sites and their free energies of OER, all the reaction steps from OH^−^ to O_2_ are downhill, implying a facile reaction. The Fe,Mn/N_6_-1 structure is also the active site of OER.

## Discussion

In summary, in order to take insight into the genuine mechanism of high effective atomic-dispersed M–N–C materials on ORR, dual-metal atomically dispersed Fe,Mn/N–C and the single-atom Fe/N–C and Mn/N–C catalyst were precisely design and prepared as model object, which uncover that the spin-state formation of 3*d* orbitals in transition metal ions is an important factor for optimizing the ORR activity. Both electron configuration features and theoretical calculation results demonstrate that the adjacent atomically dispersed Mn–N moieties can effectively activate the Fe^III^ sites and permits Fe^III^ in FeN_4_ to achieve the ideal one eg electron (t_2g_4e_g_1) filling, which can penetrate the antibonding π-orbital of oxygen easily, allowing the excellent ORR performances of Fe,Mn/N–C in both alkaline and acidic media (E_1/2_ are 0.928 V in 0.1 M KOH, and 0.804 V in 0.1 M HClO_4_). DFT theoretical calculations further confirms that the electronic structure-adjusted Fe,Mn/N–C and the oxygen intermediate have proper bond length and binding energy, which improves the reaction kinetics of ORR. As practical applications, Fe,Mn/N–C was successfully incorporated as an effective air cathode for a rechargeable flexible Zn–air battery device with long-term work stability. This work opens up new opportunities for optimizing noble-metal-free catalysts to achieve high-efficiency and stable catalysts towards fuel cells, metal–air batteries, and other renewable energy systems.

## Method

### Synthesis of Fe,Mn/N–C

Fe,Mn/N–C was synthesized via a soft-template pyrolysis method. Typically, 5 g dicyandiamide, 2 mL of phytic acid, 5 mg Iron phthalocyanine, and 5 µL 50% Manganese nitrate water solution were dissolved in 40 mL of water under stirring and dried in an oven at 80 °C for 24 h. The obtained solid powder was placed in a tubular carbonization furnace and carbonized at 900 °C for 6 h under Ar gas conditions at a heating rate of 10 °C min^−1^. The obtained sampled is named Fe,Mn/N–C.

### Synthesis of Fe/N–C and Mn/N–C

Fe/N–C and Mn/N–C were prepared using a similar process to that of Fe,Mn/N–C, except for the use of Iron phthalocyanine and 50% Manganese nitrate water solution, respectively.

### Characterizations

The morphology of the samples was characterized by transmission electron microscope (TEM, FEI Tecnai G220) with an accelerating voltage of 200 kV and field-emission scanning electron microscope (FE-SEM, JEORJSM-6700F). The HAADFSTEM images were obtained by JEOL JEM-ARM200F at an accelerating voltage of 200 kV. The crystal phases present in each sample were identified using powder X-ray diffraction(XRD) patterns were recorded on a Y-2000X-ray Diffractometer with copper Kα radiation (λ = 1.5406 Å) at 40 kV, 40 mA. The X-ray photoelectron spectroscopy (XPS) measurements were performed with an ESCA LAB 250 spectrometer on a focused monochromatic Al Kαline (1486.6 eV) X-ray beam with a diameter of 200 μm. The Raman measurements were taken on a Renishaw spectrometer at 532 nm on a Renishaw Microscope System RM2000. The N_2_ adsorption/desorption curve was carried out by BET measurements using a Micromeritics ASAP 2020 surface area analyzer. The Fe and Mn K-edge X-ray absorption near edgestructure (XANES) and the extended X-ray absorption fine structure (EXAFS) were investigated at the SXRMB beamline at the Canadian Light Source. References, such as Fe and Mn foils, are used to calibrate the beamlie energy and for comparison to samples. Fluorescence detection was performed using a 7-element Si drift detector for samples and the total electron yield was used for measurement of samples with high concentration, such as references. The EXAFS raw data were then background-subtracted, normalized and Fourier transformed by the standard procedures with the IFEFFIT package. The Mössbauer measurements were performed using a conventional spectrometer (Germany, Wissel MS-500) in transmission geometry with constant acceleration mode. A 57Co(Rh) source with activity of 25 mCi was used. The 5 velocity calibration was done with a room temperature α-Fe absorber. The spectra were fitted by the software Recoil using Lorentzian Site Analysis.

The Fe K-edge theoretical XANES calculations were carried out with the FDMNES code in the framework of real-space full multiple-scattering (FMS) scheme using Muffin-tin approximation for the potential. The energy-dependent exchange-correlation potential was calculated in the real Hedin–Lundqvist scheme, and then the spectra convoluted using a Lorentzian function with an energy-dependent width to account for the broadening due both to the core–hole width and to the final state width.

### Electrocatalytic measurement

Electrochemical experiments were conducted on a CHI760E electrochemical workstation (CH Instrument Co., USA). CV, RDE, and RRDE measurements (Pine Research Instrument, USA) were conducted using a standard three-electrode system. All the measurements were carried out at room temperature. For the preparation of working electrode, 2 mg of catalyst was dispersed in 1 mL mixture of ethanol and 5% Nafion (v:v = 200:1) under sonication for 1 h to form a homogeneous catalyst ink. Then 10 μL of this catalyst ink was loaded onto a glassy carbon rotating disk electrode with the diameter of 5 mm, resulting in the catalyst loading of 0.1 mg cm^−2^, followed by drying at room temperature.

For the ORR at an RDE, the working electrode was scanned cathodically at a rate of 5 mV s^−1^ with varying rotating speed from 400 to 2250 rpm in O_2_-saturated 0.1 M KOH aqueous solution. The electron transfer number per oxygen molecule for oxygen reduction can be determined on the basis of the Koutechy–Levich equation (ref):

3 $$\frac{1}{J} = \frac{1}{{J_{\mathrm{L}}}} + \frac{1}{{J_{\mathrm{k}}}} = \frac{1}{{B\omega ^{0.5}}} + \frac{1}{{J_{\mathrm{k}}}}$$

4 $${\mathrm{B}} = 0.62nFC_0(D_0)^{2/3}v^{ - 1/6}$$

5 $$J_{\mathrm{k}} = nFkC_0$$

Where *J* is the measured current density and is the electrode rotating rate (rad s^−1^). *B* is determined from the slope of the Koutechy–Levich (K-L) plot based on Levich equation. *J*_L_ and *J*_K_ are the diffusion- and kinetic-limiting current densities, *n* is the transferred electron number, *F* is the Faraday constant (*F* = 96485 C mol^−1^), *C*_0_ is the O_2_ concentration in the electrolyte (*C*_0_ = 1.26 × 10^−6^ mol cm^−3^), *D*_0_ is the diffusion coefficient of O_2_ (*D*_0_ = 1.93 × 10^−5^ cm^2^ s^−1^), and *v* is the kinetic viscosity (*v* = 0.01009 cm^2^ s^−1^). The constant 0.62 is adopted when the rotation speed is expressed in rad s^−1^.

For the RRDE measurements, the disk electrode was scanned cathodically at a rate of 10 mV s^−1^ and the ring potential was kept at 1.5 V versus RHE. The peroxide percentage and the electron transfer number (*n*) were determined by the following equations (ref):

6 $${\mathrm{HO}}_2^ - = 200 \times \frac{{I_{\mathrm{R}}/N}}{{I_{\mathrm{D}} + I_{\mathrm{R}}/N}}$$

7 $${\mathrm{n}} = 4 \times \frac{{I_{\mathrm{D}}}}{{I_{\mathrm{D}} + I_{\mathrm{R}}/N}}$$

where *I*_d_ is disk current, Ir is ring current, and *N* is current collection efficiency of the Pt ring. *N* was determined to be 0.40.

#### All-solid-state Zn–air battery assembly

A polished zinc foil (0.05 mm thickness) was used as anode. The gel polymer electrolyte was prepared as follow: polyvinyl alcohol (PVA, 5 g) was dissolved in 50 mL was added 18 M KOH (5 mL) at 95 °C to form a homogeneous viscous solution, followed by casting on a glass disk to form a thin polymer film (thickness about 2 mm). The film was then freezed in a freezer at −20 °C about 2 h, and then keep at 0 °C temperature about 4 h. The film was thawed for 12 h before used. Then, the as-prepared Fe,Mn/N–C film and zinc foil were placed on the two sides of PVA gel, followed by pressed Ni foam as current collector. The components were firmly pressed together by roll-pressing. No inert atmosphere or glove-box is required for the packaging.

#### Zinc–air battery tests

The catalyst ink recipe consists of 5.0 mg catalyst dispersed in 480 μL of DI water/isopropyl alcohol (v/v~3:7)/20 μL Nafion (5 wt.%) solution. For the Zn–air battery test, the air electrode was prepared by uniformly coating the as-prepared catalyst ink onto carbon paper then drying it at 80 °C for 2 h. The mass loading was 0.5 mg cm^−2^ unless otherwise noted. A Zn plate was used as the anode and catalysis loaded on carbon paper are used as cathodes. Both electrodes were assembled into a home-made Zn–air battery, and 6 M KOH aqueous solutions was used as the electrolyte^28^. All Zn–air batteries were evaluated under ambient conditions. The polarization curves were recorded by linear sweep voltammetry (5 mV s^−1^, at room temperature) on a CHI 760D electrochemical platform.

### Computation methods

First-Principles calculations were carried out within the density functional theory framework. The projector-augmented wave (PAW) method and the generalized gradient approximation (GGA) for the exchange-correlation energy functional, as implemented in the Vienna ab initio simulation package (VASP) were used. The GGA calculation was performed with the Perdew–Burke–Ernzerhof (PBE) exchange-correlation potential. A plane-wave cutoff energy of 400 eV was used. All atoms were fully relaxed with a tolerance in total energy of 0.1 mV, and the forces on each atom were less than 0.01 eV/Å. All calculations were spin-polarized.

A 6x6 graphene supercell (14.807 x 14.807 Å) with 12 Å vacuum layer was first constructed, and then Fe (or Mn)-N4 structure was simulated based on this model. Fe and Mn co-doped structure was then constructed. Three co-doped type was considered. A 4 x 4 K-points was used in all these calculations.

The ORR performed on Fe or Mn site was calculated by the following theory. The four-electron pathway by which the ORR occurs under base condition are generally reported to proceed according to the following steps:

8 $${\mathrm{O}}_2\left( {\mathrm{g}} \right) + \ast \Rightarrow {\mathrm{O}}_2^ \ast $$

9 $${\mathrm{O}}_2^ \ast + {\mathrm{H}}_2{\mathrm{O}}(l) + e^ - \Rightarrow {\mathrm{OOH}}^ \ast + {\mathrm{OH}}^ - $$

10 $${\mathrm{OOH}}^ \ast + e^ - \Rightarrow {\mathrm{O}}^ \ast + {\mathrm{OH}}^ - $$

11 $${\mathrm{O}}^ \ast + {\mathrm{H}}_2{\mathrm{O}}\left( l \right) + e^ - \Rightarrow {\mathrm{OH}}^ \ast + {\mathrm{OH}}^ - $$

12 $${\mathrm{OH}}^ \ast + e^ - \Rightarrow {\mathrm{OH}}^ - + \ast $$

where * represents an active site on the corresponding surface.

The adsorption energy (ΔE_ads_) for ORR was calculated as:

13 $$E_{{\mathrm{ads}}} = E_{{\mathrm{substrate}} + {\mathrm{adsorbate}}} - E_{{\mathrm{substrate}}} - E_{{\mathrm{adsorbate}}}$$

The adsorption free energy (ΔG_ads_) is obtained by

14 $$\Delta G_{{\mathrm{ads}}} = \Delta E + \Delta ZPE - T\Delta S + \Delta G_{\mathrm{U}} + \Delta G_{{\mathrm{pH}}}$$

where Δ*E* is the energy difference of reactants and products, obtained from DFT calculations; ΔZPE and ΔS are the contributions to the free energy from the zero-point vibration energy and entropy, respectively. *T* is the temperature (300 K). Δ*G*_*U*_ = −*eU*, here *U* is the potential at the electrode and *e* is the transferred charge. Δ*G*_pH_ is the correction of the H^+^ free energy. The free energy of H^+^ ions has been corrected by the concentration dependence of the entropy:

15 $$G\left( {{\mathrm{pH}}} \right) = - kT\,{\mathrm{ln}}\,\left[ {H^ + } \right] = kT{\mathrm{ln10}} \ast {\mathrm{pH}}$$

(0.059526 for 0.1 M HClO_4_; 0.773844 for 0.1 M KOH).

## Supplementary information

Supplementary Information

## Data Availability

The data underlying Figs. 1–5, Supplementary Figs. 2–10, 12–29 and 31 are provided as a Source Data file. The other data support the findings of this study are available from the corresponding author upon request. Source data are provided with this paper.

## Source data

Source Data
