# Supplementary material for: Regulating Fe-spin state by atomically dispersed Mn-N in Fe-N-C catalysts with high oxygen reduction activity
Source: Nat Commun. 2021 Mar 19;12:1734. doi: 10.1038/s41467-021-21919-5 (PMC7979714; doi:10.1038/s41467-021-21919-5)
Supplement: Supplementary file 1 — Supplementary Information [file 41467_2021_21919_MOESM1_ESM.pdf]

Supplementary Information

Regulating Fe-Spin State by Atomically Dispersed Mn-N in Fe-N-C Catalysts with High Oxygen Reduction Activity

Gege Yang,[1] Jiawei Zhu,[2,5] Pengfei Yuan,[3] Yongfeng Hu,[4] Gan Qu,[1] Bang-An Lu,[1] Xiaoyi Xue,[1] Hengbo Yin,[1] Wenzheng Cheng,[1] Junqi Cheng,[1] Wenjing Xu,[1] Jin Li,[1] Jinsong Hu,[6] Shichun Mu[,2,5] and Jianan Zhang,*[,1]*

*correspondence to: msc@whut.edu.cn; zjn@zzu.edu.cn

Supplementary Figures and Tables

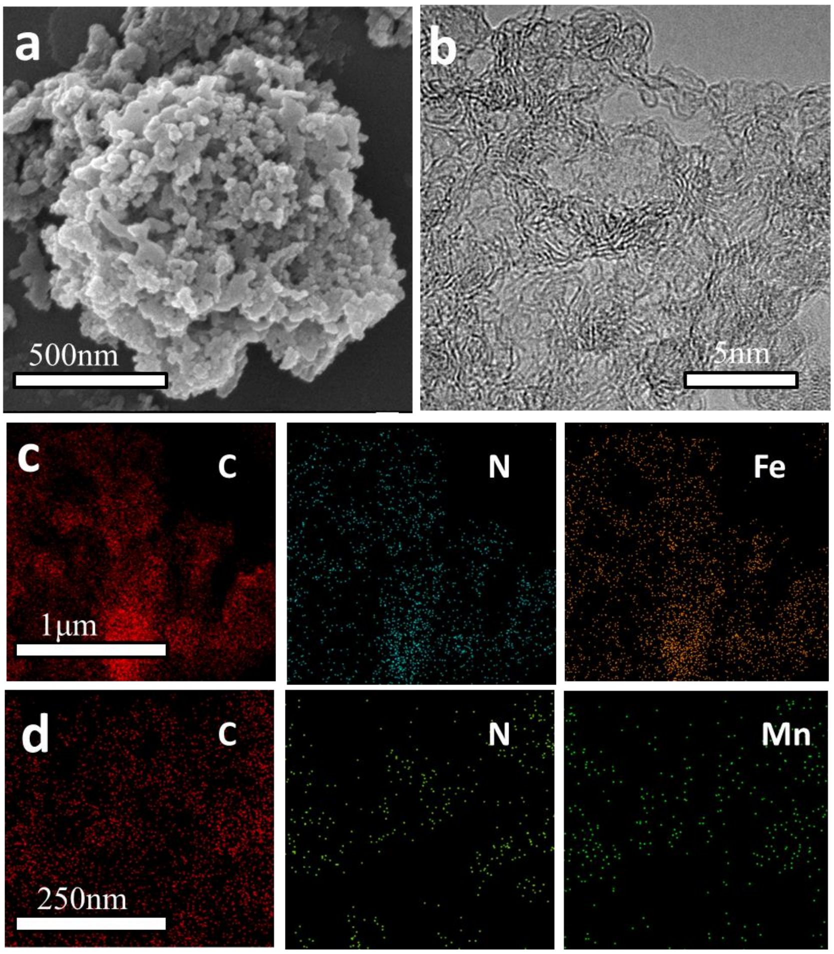

Supplementary Figure 1: SEM (a) and TEM (b) images of FeMn/N-C. EDS-mapping images of (c) Fe/N-C (d) Mn/N-C

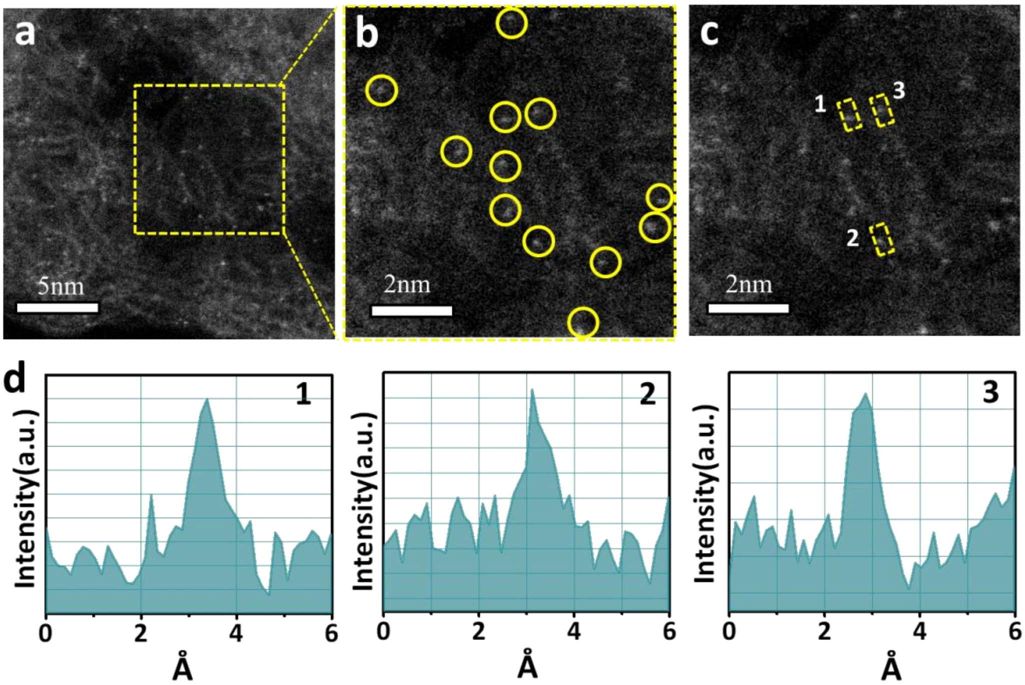

Supplementary Figure 2: (a) and (b) HAADF-STEM image of Fe/N-C. (c) and (d) Several single atoms have been highlighted and different intensity profiles have been obtained on them.

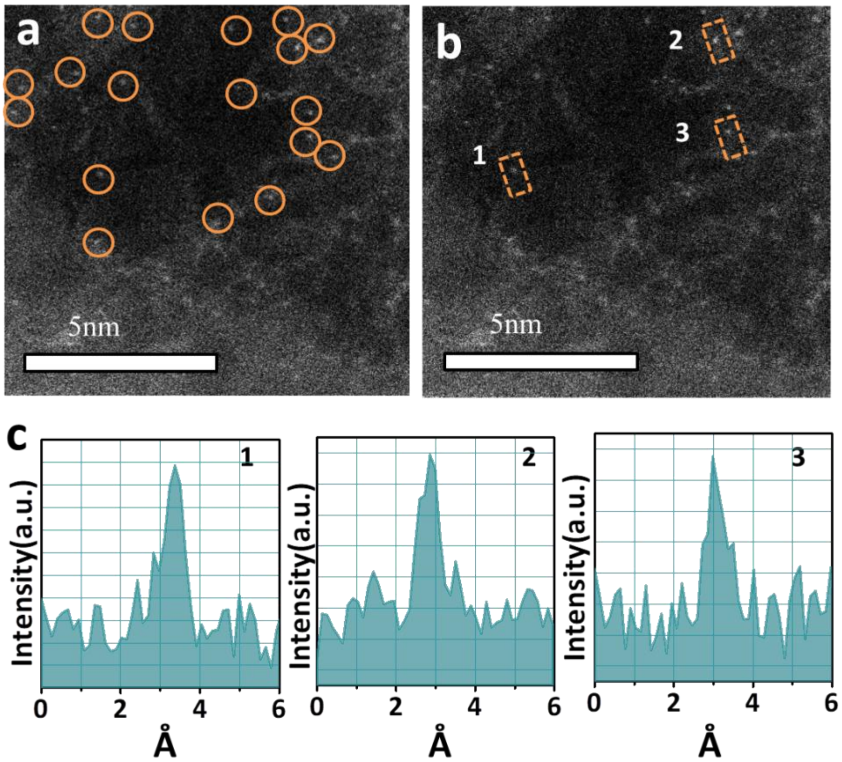

Supplementary Figure 3: (a) HAADF-STEM image of Mn/N-C. (b) and (c) Several single atoms have been highlighted and different intensity profiles have been obtained on them.

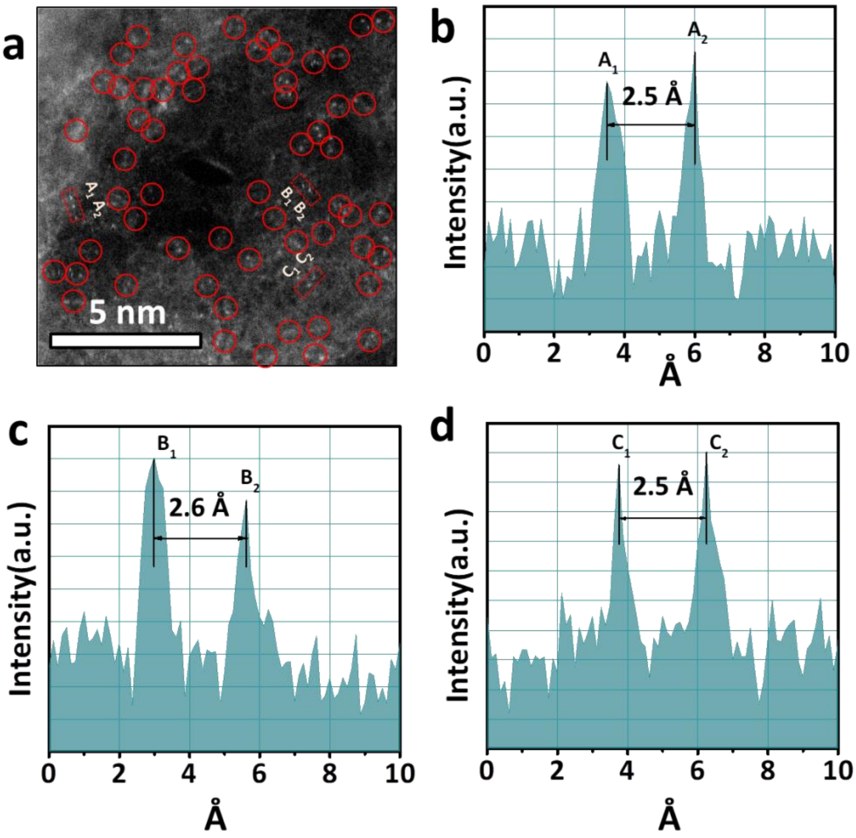

Supplementary Figure 4: (a-d) Several Fe-Mn dual atoms have been highlighted and different intensity profiles have been obtained on them

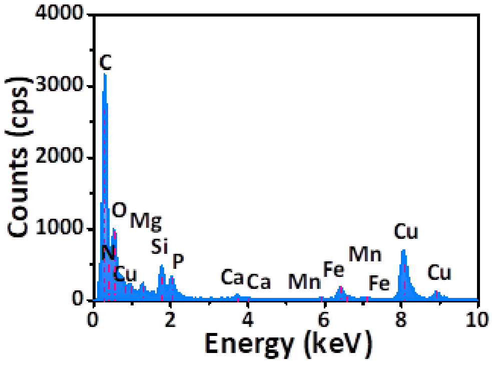

Supplementary Figure 5: Energy dispersive X-ray Spectroscopy image

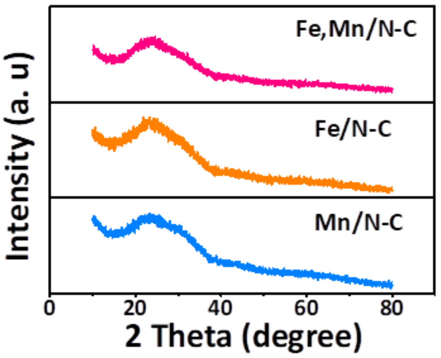

Supplementary Figure 6: XRD pattern of Fe,Mn/N-C, Fe/N-C and Mn/N-C

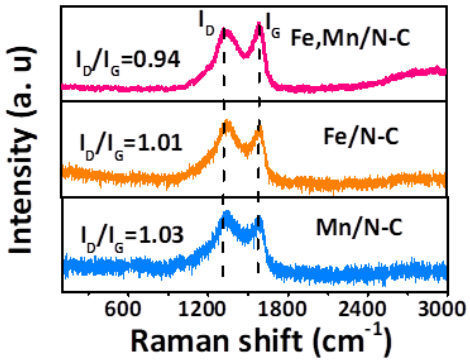

Supplementary Figure 7: Raman spectra of different samples

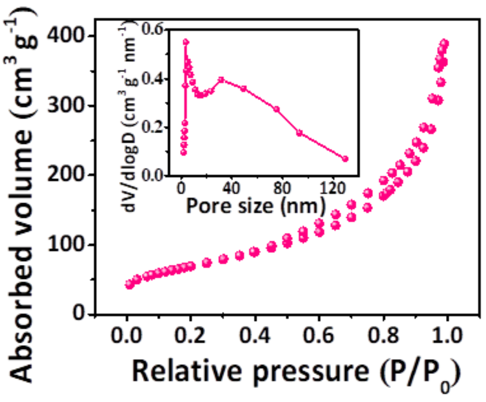

Supplementary Figure 8:N_2 adsorption/desorption isotherms of Fe,Mn/N-C, inset is pore size distribution of the Fe,Mn/N-C.

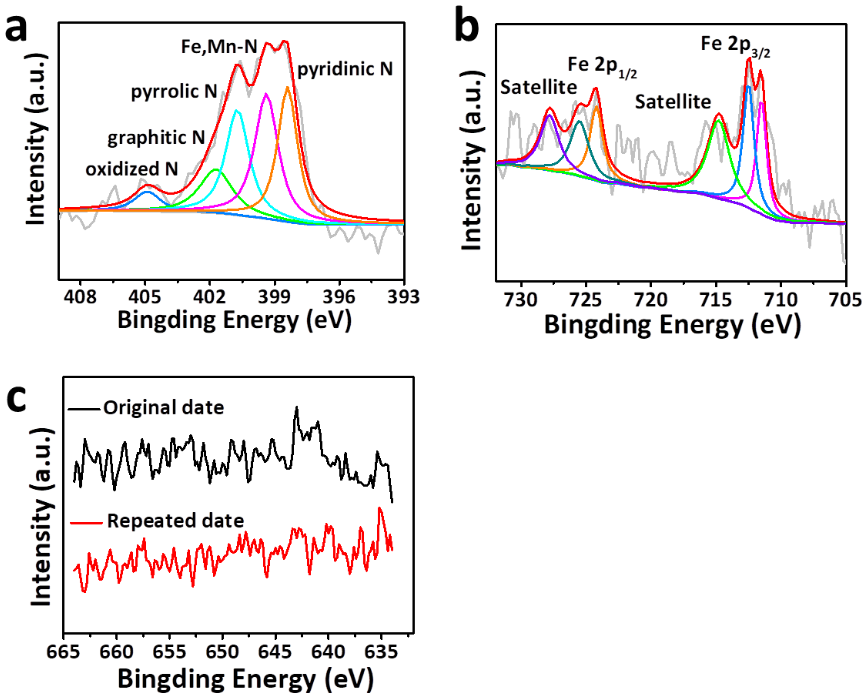

Supplementary Figure 9: (a) High-resolution N 1s spectrum of Fe,Mn/N-C, High-resolution XPS spectra for Fe 2p (b) and Mn 2p (c) for Fe,Mn/N-C.

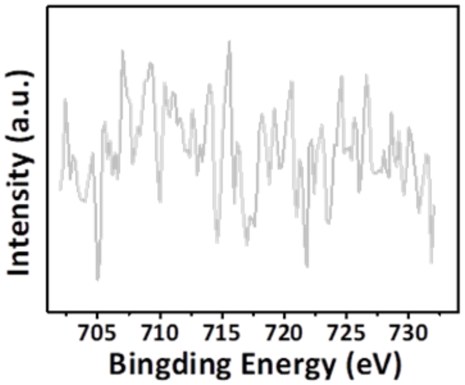

Supplementary Figure 10: High-resolution XPS spectra for Fe 2p for Fe/N-C.

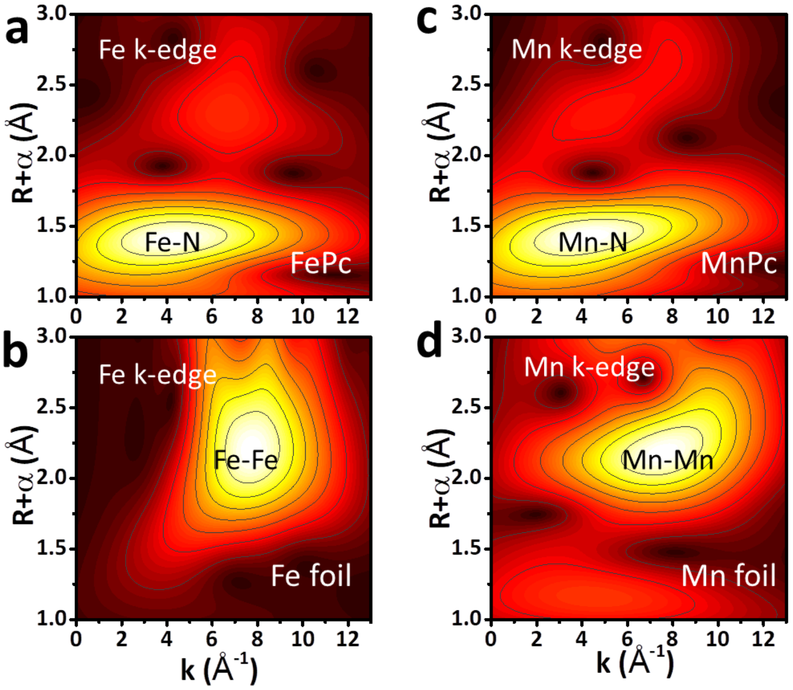

Supplementary Figure 11: Wavelet transform of the k^3-weighted EXAFS data of (a) FePc , Fe foil and (b)MnPc , Mn foil.

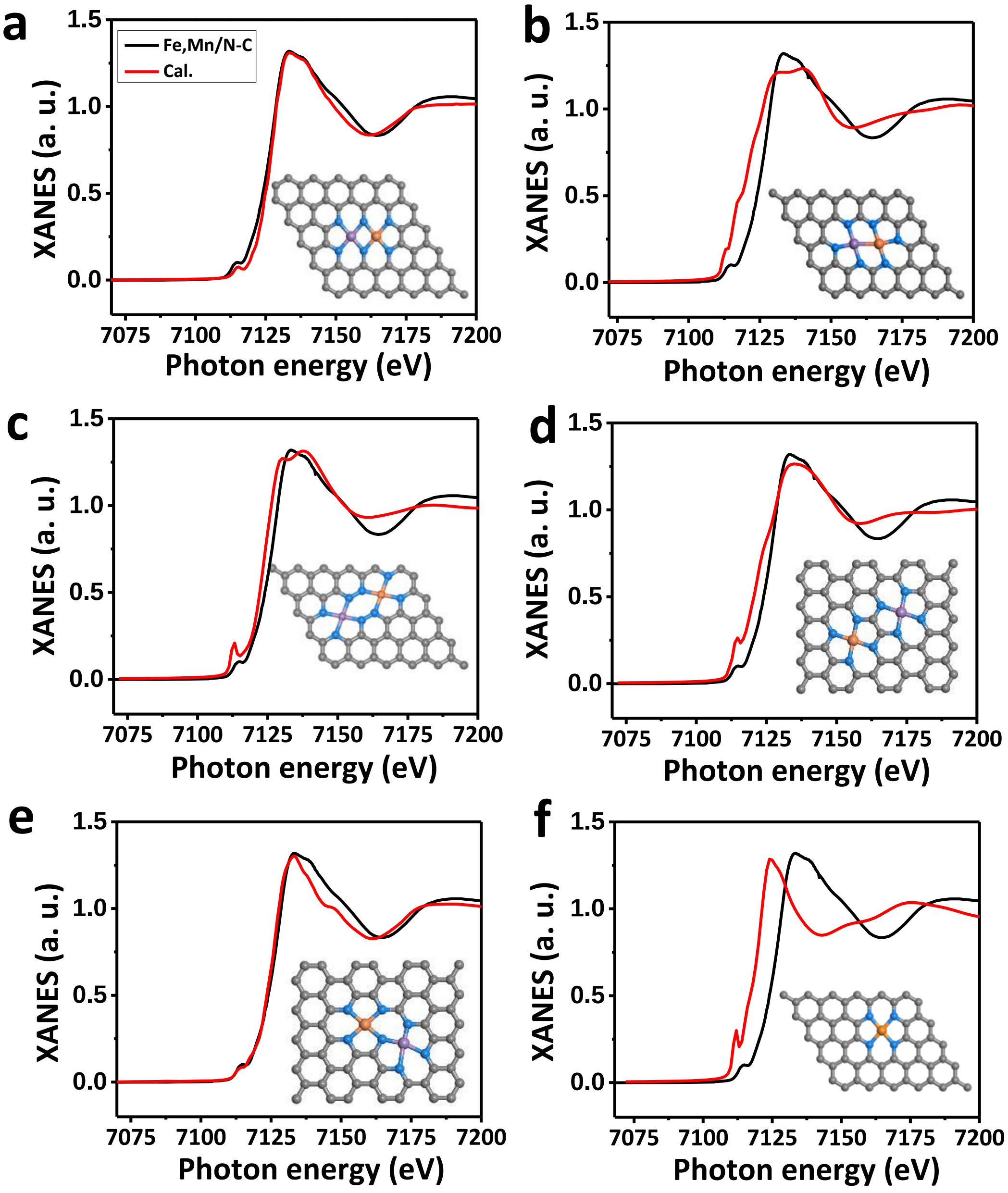

Supplementary Figure 12: Comparison between the K-edge XANES experimental spectra of Fe,Mn/N-C (black lines) and the theoretical spectra calculated with the depicted structures (red lines).

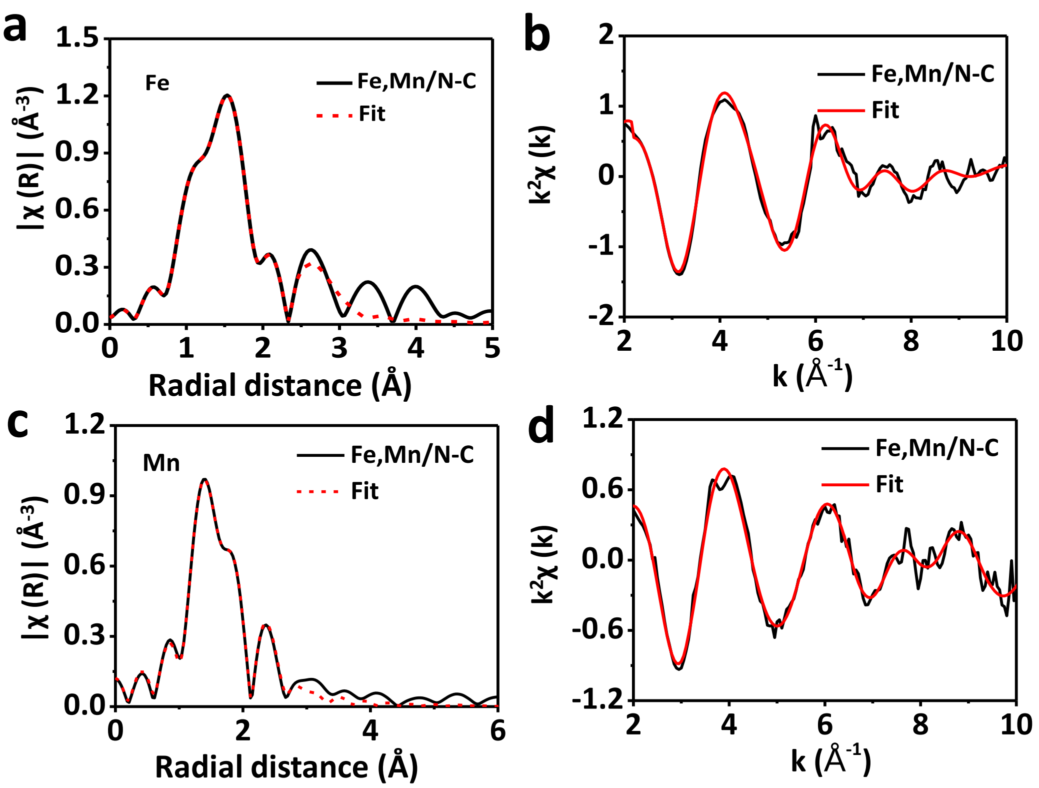

Supplementary Figure 13: (a, b) The Fe k-edge Fourier-transform EXAFS (a) and k space curves (b) of Fe,Mn/N-C and corresponding fitting curves. (c,d) The Mn k-edge Fourier-transform EXAFS (c) and k space curves (d) of of Fe,Mn/N-C and corresponding fitting curves

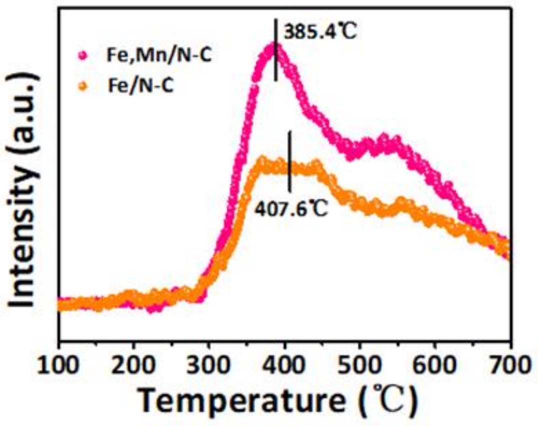

Supplementary Figure 14: O_2-TPD of Fe/N-C and Fe,Mn/N-C.

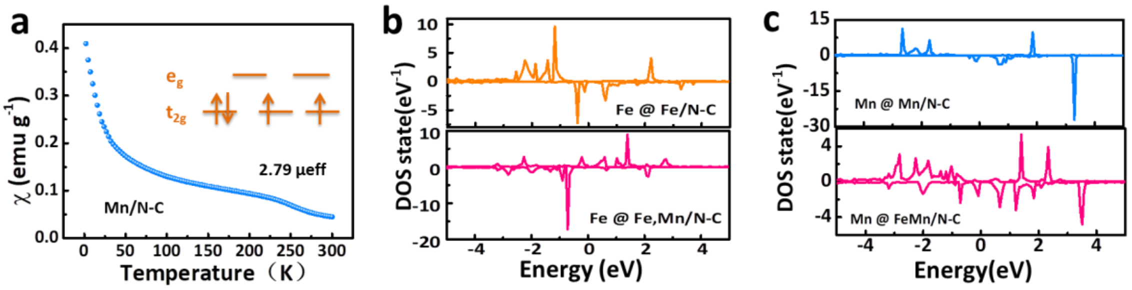

Supplementary Figure 15: (a) Magnetic susceptibility of Mn/N-C. (b) DOS of Fe@Fe/N-C and Fe@Fe,Mn/N-C. (c) DOS of Mn@Mn/N-C and Mn@Fe,Mn/N-C.

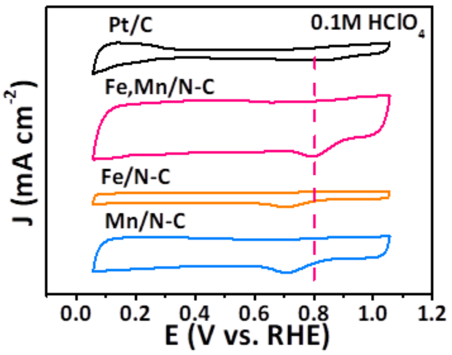

Supplementary Figure 16: CVs of Fe, Mn/N-C, Fe/N-C, Mn/N-C and Pt/C for ORR catalysis in 0.1M $HClO_4$.

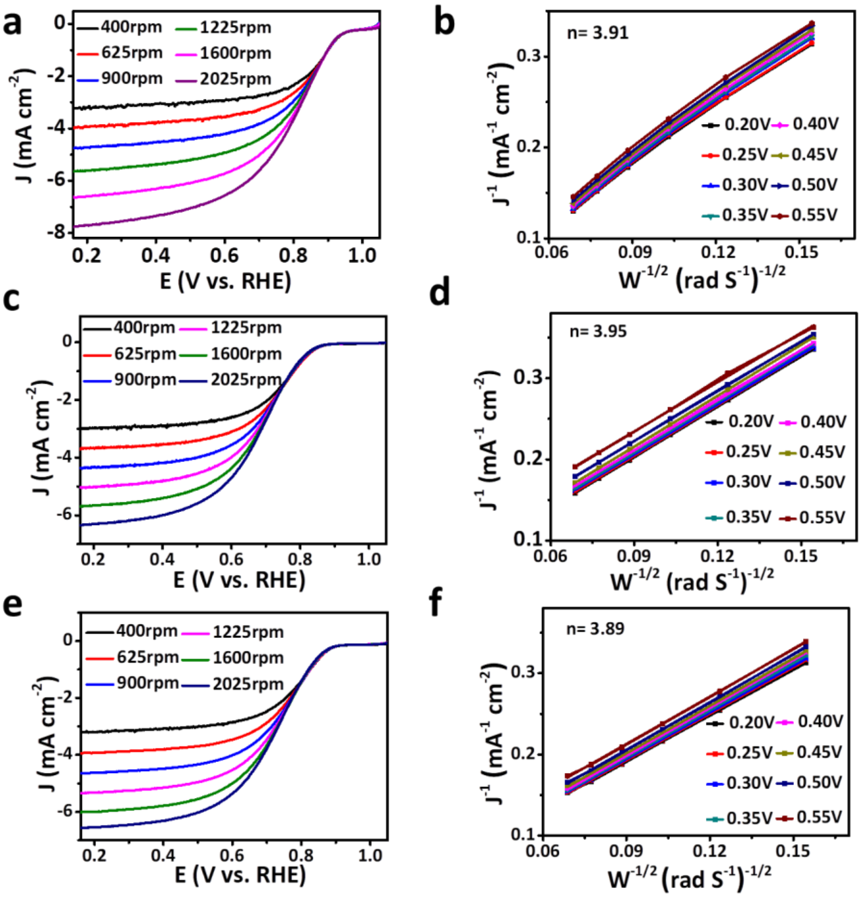

Supplementary Figure 17: LSV curves and K-L plots of (a. b) Fe,Mn/N-C, (c. d) Fe/N-C, (e. f) Mn/N-C in O_2-saturated 0.1 M $HClO_4$ solution.

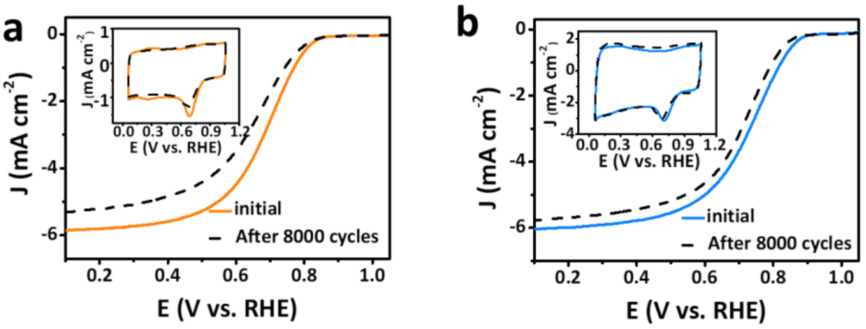

Supplementary Figure 18: ORR polarization LSV and CV curves of Fe/N-C (a) and Mn/N-C (b), measurement before and after 8000 potential cycles at the scan rate of 50 mV s^{-1} with the rotation speed of 1600 rpm in 0.1M $HClO_4$.

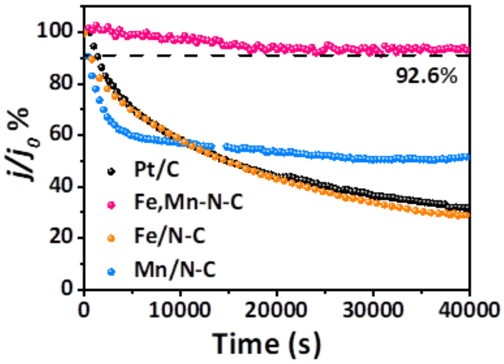

Supplementary Figure 19: The chronoamperometric response of Fe,Mn/N-C, Fe/N-C, Mn/N-C and Pt/C in an O_2-saturated 0.1 M $HClO_4$ solution at a potential of 0.6 V.

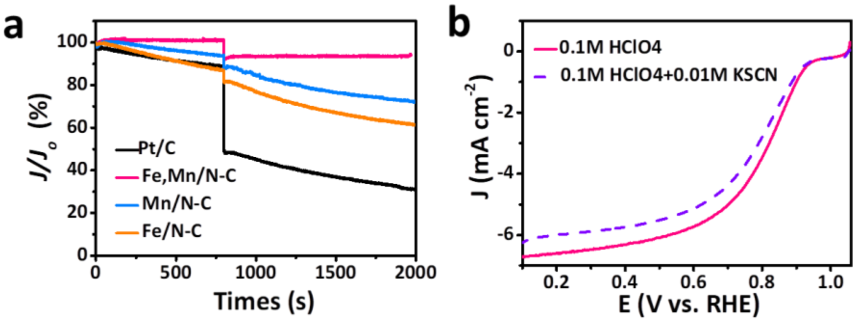

Supplementary Figure 20: (a) Chronoamperometric response for Fe,Mn/N-C, Fe/N-C, Mn/N-C and Pt/C electrode at 0.6 V (vs. RHE) after the introduction of 9.7 ml of CH_3OH into 230.3 ml of 0.1M $HClO_4$ solution. (b) Poisoning experiments of Fe,Mn/N-C measured by RDE in an O_2-saturated 0.1 M $HClO_4$ electrolyte with and without KSCN in the electrolyte.

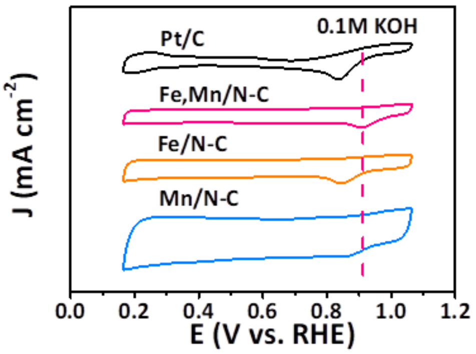

Supplementary Figure 21: CVs of Fe,Mn/N-C, Fe/N-C, Mn/N-C and Pt/C for ORR catalysis in 0.1M KOH.

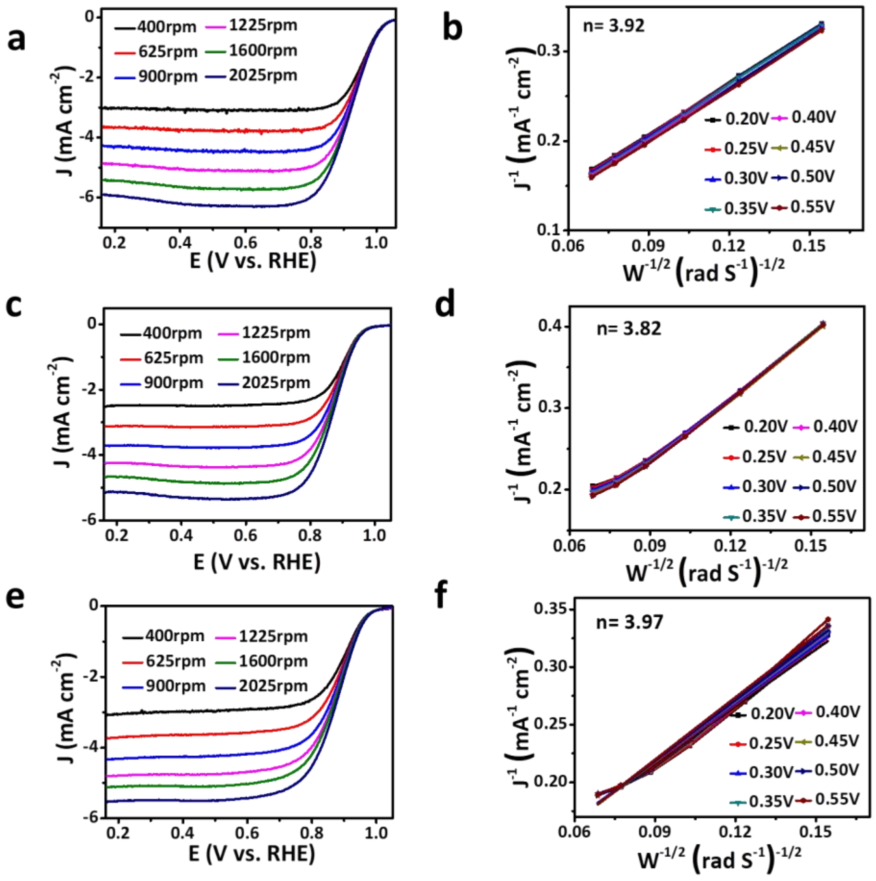

Supplementary Figure 22: LSV curves and K-L plots of (a. b) Fe,Mn/N-C, (c. d) Fe/N-C, (e. f) Mn/N-C in O_2-saturated 0.1 M KOH solution.

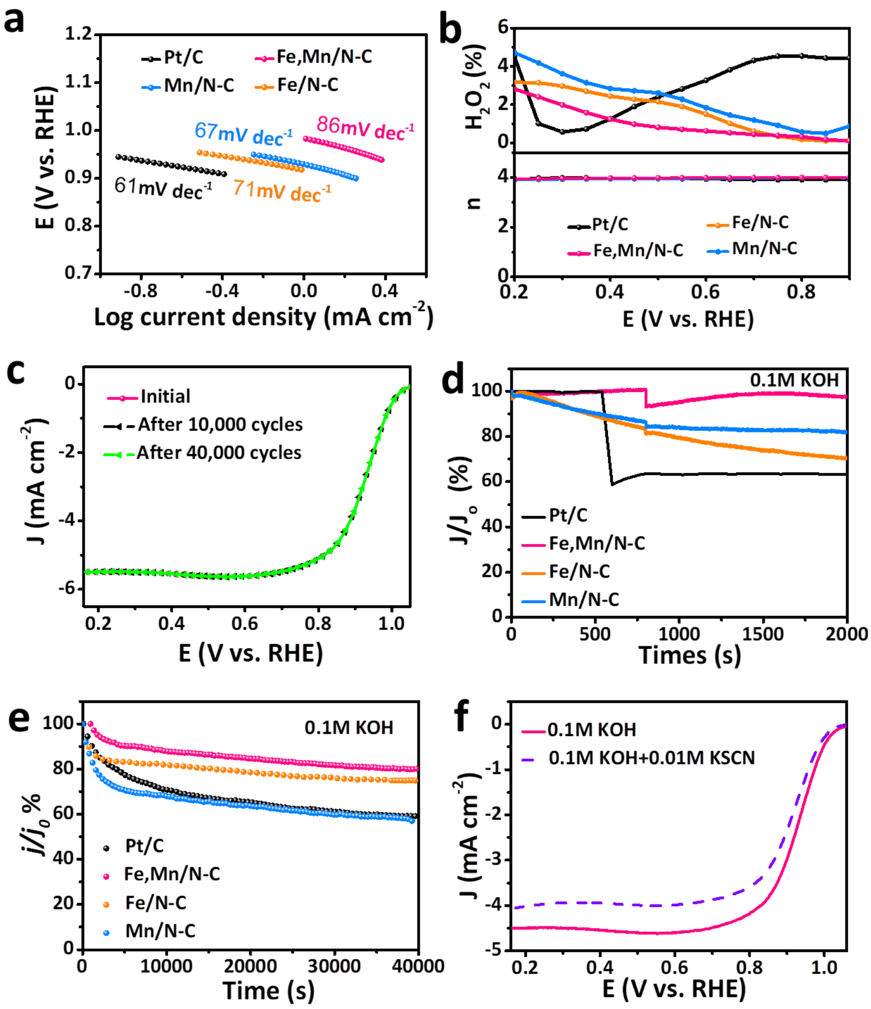

Supplementary Figure 23: (a) Corresponding Tafel plots obtained from the RDE polarization curves (b) H_2O_2 yield and electron transfer number .(c) ORR polarization of Fe,Mn/N-C measurement before and after 10000 and 40000 potential cycles at the scan rate of 50 mV s^{-1} with the rotation speed of 1600 rpm. (d) The chronoamperometric response of Fe,Mn/N-C, Fe/N-C, Mn/N-C and Pt/C in an O_2-saturated 0.1 M KOH solution at a potential of 0.6V. (e)Chronoamperometric response for Fe,Mn/N-C, Fe/N-C, Mn/N-C and Pt/C electrode at 0.6 V (vs. RHE) after the introduction of 9.7 ml of CH_3OH into 230.3 ml of 0.1M KOH solution.(f)Poisoning experiments of Fe,Mn/N-C measured by RDE in an O_2-saturated 0.1 M $HClO_4$ electrolyte with and without KSCN in the electrolyte

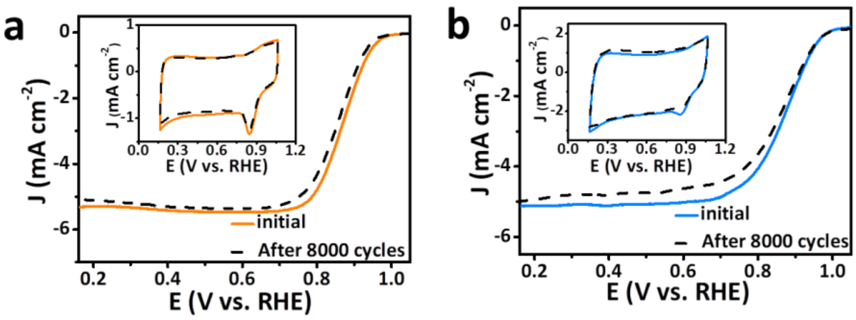

Supplementary Figure 24: ORR polarization LSV and CV curves of (a) Fe/N-C and (b) Mn/N-C, measurement before and after 8000 potential cycles at the scan rate of 50 mV s^{-1} with the rotation speed of 1600 rpm in 0.1M KOH.

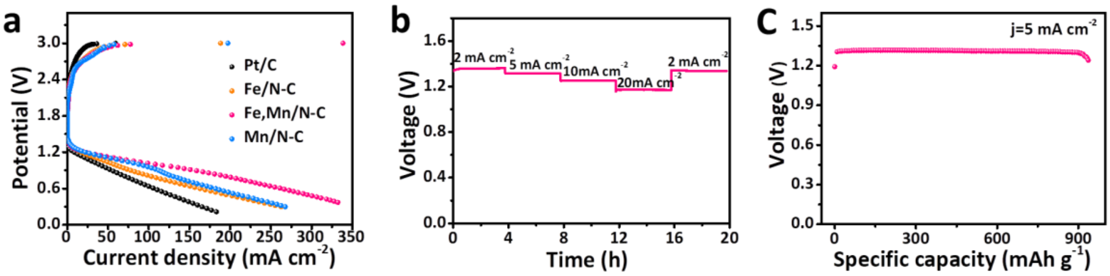

Supplementary Figure 25: (a) Charge and discharge polarization curves of Zn–air batteries. (b) Galvanostatic discharge curves of the primary Zn-air battery with Fe,Mn/N-C as catalyst at different current densities, which was normalized to the area of air-cathode. (c) Galvanostatic discharge curves of the primary Zn-air battery with Fe,Mn/N-C as catalyst at 5 mA cm^{-2} current densities.

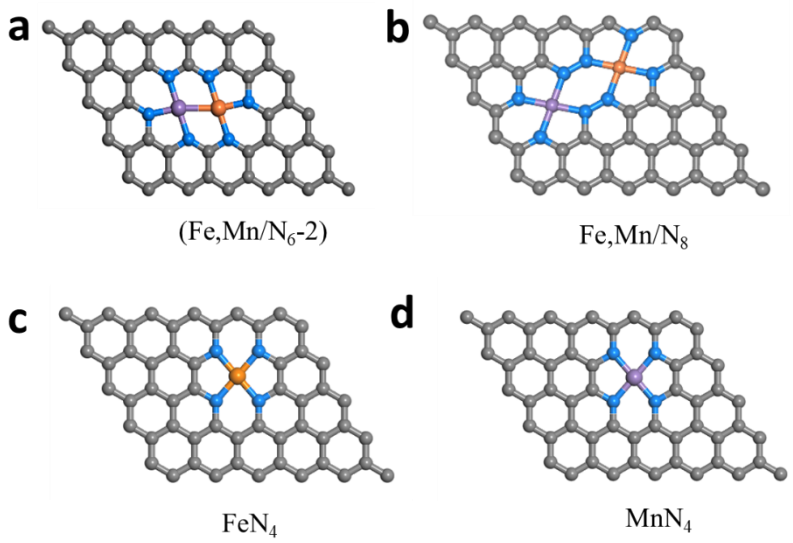

Supplementary Figure 26: The optimized structure of different models: Fe,Mn/N_6-2(a), Fe,Mn/N_8(b), FeN_4(c), MnN_4(d).

Fe,Mn/N_8 includes two models, Fe,Mn/N_8-a represents ORR active site is Fe, Fe,Mn/N_8-b represents ORR active site is Mn.

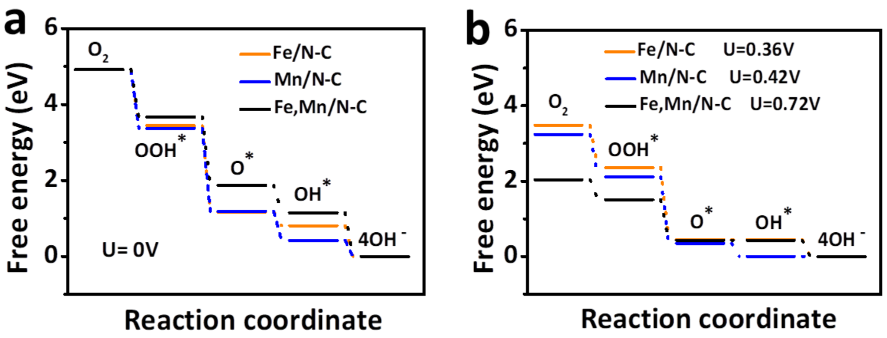

Supplementary Figure 27: (a) The Gibbs free-energy path pathways of both four-electron based alkaline ORR at U = 0 V. (b) The pathways for Fe/N-C, Mn/N-C and Fe,Mn/N-C are summarized at U = 0.36 V, 0.42 V, and 0.72 V, respectively.

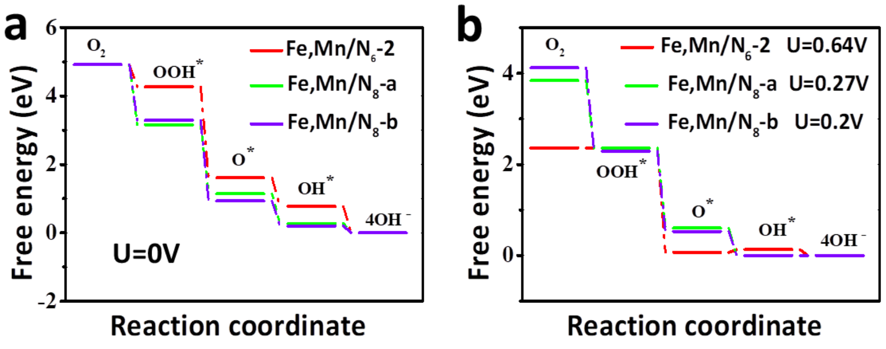

Supplementary Figure 28: Free-energy path of ORR for Fe,Mn/N_6-2 , Fe,Mn/N_8-a and Fe,Mn/N_8-b in an alkaline solution.

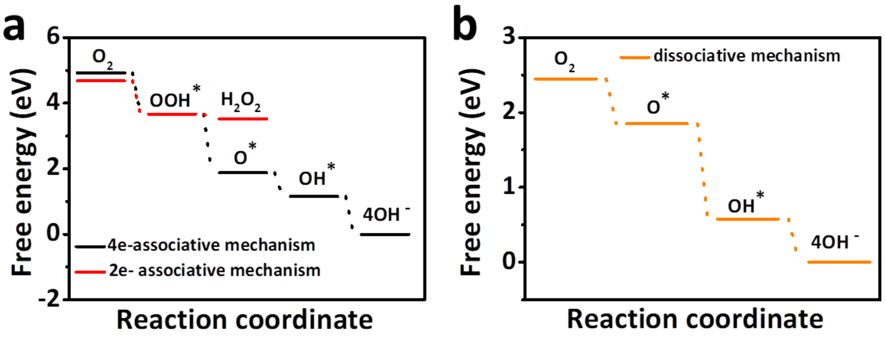

Supplementary Figure 29: Free energy diagrams for associative and dissociative mechanisms of Fe,Mn/N6-1.

We have considered different reaction pathway (4e- associative mechanism, 2e- associative mechanism and dissociative mechanism) in our pre-calculations. Take Fe,Mn/N_6-1 as an example, we have made different free energy diagrams for different kinetic mechanisms, and accordingly calculated the theoretical overpotentials. It can be seen that among them, the 4e- associative mechanism possesses the smallest overpotential (0.511 V vs. 0.552 V for 2e- associative mechanism and 0.656 V for dissociative mechanism), namely, it is more likely to happen from the point of thermodynamics. Thus, we only display the most common 4e- associative mechanism in our manuscript.

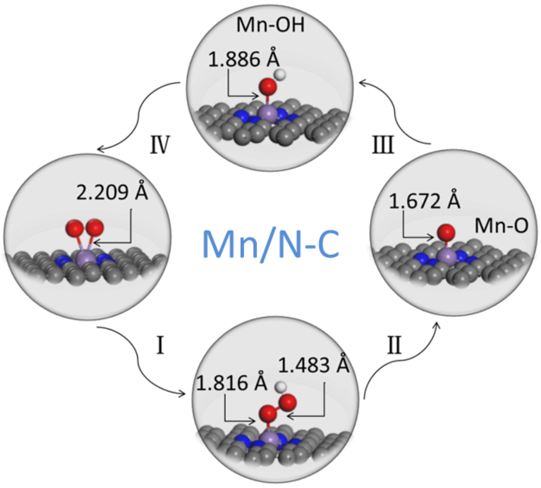

Supplementary Figure 30: Optimized atomic structures for the main process of an ORR: Mn/N-C

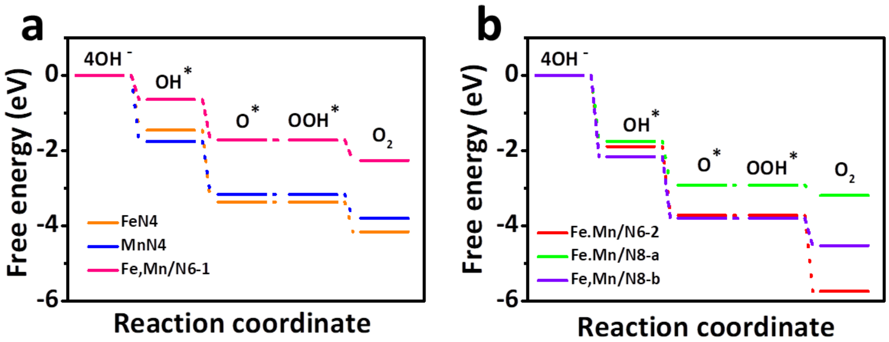

Supplementary Figure 31: The Gibbs free-energy path pathways of both four-electron based alkaline OER.

Supplementary Tables

Supplementary Table 1. Raman results analysis for the prepared samples .

**Sample Name**	**Fe,Mn/N-C**	**Fe/N-C**	**Mn/N-C**
I_D/I_G	0.94	1.01	1.03

Supplementary Table 2. XPS results analysis for the prepared samples (at. %).

**Sample Name**	**C (at. %)**	**N (at. %)**	**O (at. %)**	**Fe (at. %)**	**Mn (at. %)**
Fe,Mn / N-C	79.8	8.66	9.82	0.06	0.05
Fe / N-C	80.55	4.73	12.98	0.36	0
Mn/N-C	74.16	9.94	14.06	0	0.15

Supplementary Table 3. XPS results analysis of high-resolution N 1s spectrum for the prepared samples (at. %).

**Sample Name**	**pyridinic-N (at. %)**	**pyrrolic-N (at. %)**	**graphitic-N (at. %)**	**pyridinic N^+-O^- (at. %)**	**metal-N (at. %)**
**Fe,Mn / N-C**	33.10	26.16	17.76	6.81	16.17
**Fe / N-C**	29.22	24.68	14.26	5.13	26.71
**Mn / N-C**	33.36	27.16	12.72	5.95	20.77

Supplementary Table 4. EXAFS fitting parameters at the Mn and Fe K-edge various samples.

Sample	Scattering pair	CN	R(Å)	σ2(10-3Å2)	R factor
Fe in Fe,Mn/N-C	Fe-N1	1.8±0.3	1.97±0.02	5.7±0.5	0.005
	Fe-N2	2.0±0.4	2.02±0.02	6.2±0.7	
	Fe-Mn	0.9±0.2	2.59±0.03	6.8±0.5	
Mn in Fe,Mn/N-C	Mn-N1	1.9±0.3	1.95±0.02	5.3±0.4	0.008
	Mn-N2	2.2±0.4	2.00±0.02	5.9±0.6	
	Mn-Fe	1.1±0.2	2.62±0.03	6.5±0.7	

$S0^2$ is the amplitude reduction factor; CN is the coordination number; R is interatomic distance (the bond length between central atoms and surrounding coordination atoms); σ^2 is Debye-Waller factor (a measure of thermal and static disorder in absorber-scatter distances). R factor is used to value the goodness of the fitting.

Error bounds that characterize the structural parameters obtained by EXAFS spectroscopy were estimated as N ± 20%; R ± 1%; σ^2 ± 20%; ΔE_0 ± 20%.

Supplementary Table 5. Summary of the Mossbauer parameters and assignments to different Iron species in Fe,Mn/N-C.

Fe species	IS (mm $s^{-1)}$	QS (mm s^{-1})	Area	Content (%)
D1	0.993	3.27	8700	13.5
D3	1.058	2.09	17300	27.1
D4	0.312	0.654	38000	59.4

Supplementary Table 6. Comparison sample of the as-prepared Fe,Mn/N-C electrocatalysts in 0.1M $HCIO_4$.

**Catalysts**	**Onset Potential (V)**	**E½ (V vs. RHE)**	**$J_{Limiting}$ (mA cm^{-2})**	**J_K (mA cm^{-2})**
**Fe,Mn /N-C**	0.989	0.804	6.77	7.75
**Fe /N-C**	0.778	0.702	5.76	6.29
**Mn /N-C**	0.823	0.730	6.07	7.13
**Pt/C**	0.866	0.807	5.90	7.22

Supplementary Table 7. Comparison of ORR catalytic activity between Fe,Mn/N-Cproduct and other well-developed Carbon based ORR electrocatalysts in acidic solution.

Catalysts	electrolyte	$E_{1/2}$(V)	$J_{limiting}$ (mA cm^{-2})	Reference
Fe,Mn/N-C	0.1M $HClO_4$	0.804	6.77	This work
Mn-N_4	0.5M H_2SO_4	0.8	＜5	1
Fe_{SA}-N-C	0.1M $HClO_4$	0.776	5.90	2
FePhen@MOF	0.1M $HClO_4$	0.77	＜6	3
Fe_2-N-C	0.5M H_2SO_4	0.78	＜6	4
Cu@Fe-N-C	0.5M H_2SO_4	0.761	5.2	5
Fe/OES	0.5M H_2SO_4	0.72	5.5	6
Fe-N-C-1	0.1M $HClO_4$	0.74	5.0	7
Zn/Fe-N-C	0.1M $HClO_4$	0.81	5.8	8
Fe SAs/N−C	0.1M $HClO_4$	0.798	＜6	9

Supplementary Table 8. Comparison sample of the as-prepared Fe,Mn/N-Celectrocatalysts in 0.1 M KOH.

Catalysts	Onset Potential (V)	E½ (V vs. RHE)	$J_{Limiting}$ (mA cm^{-2})	J_K (mA cm^{-2})
**Fe,Mn/N-C**	0.979	0.928	5.55	7.04
**Fe/N-C**	0.917	0.872	5.41	5.74
**Mn/N-C**	0.929	0.876	4.68	6.60
**Pt/C**	0.894	0.831	5.12	7.19

Supplementary Table 9. Comparison of ORR catalystic activity between Fe,Mn/N-C product and other well-developed Carbon based ORR electrocatalysts in alkaline solution.

Catalysts	$E_{1/2}$(V)	$J_{limiting}$ (mA cm^{-2})	Reference
Fe,Mn/N-C	0.928	5.55	This work
Mn/Fe-HIB-MOF	0.883	6.37	10
Fe_{SA}-N-C	0.891	6.0	2
Zn/Fe-N-C	91	5.9	8
Fe-N_4 SAs/NPC	0.885	5.5	11
Mn/C-N	0.86	5.3	12
Fe SAC/N-C	0.89	5.6	13
Fe-ISAs/CN	0.90	6.1	14
Fe SAs/N–C	0.91	5.5	15
SA-Fe-NHPC	0.93	6.0	16
Fe_1-HNC-500-850	0.85	5.8	17

Supplementary Table 10. The formation energy for the structural models.

Model	MnN4	FeN4	Fe,Mn/N6-1	Fe,Mn/N6-2	Fe,Mn/N8
Formation energy(eV)	-3.38	-3.76	-8.13	-5.60	-5.29

Supplementary Table 11. The calculated total energy (eV) of different supports and O, OH, OOH adsorbed on different active sites.

Active sites	E (support)	E(OH*)	E(O*)	E(OOH*)
MnN_4	-654.515	-665.1460989	-660.74227269	-669.62659395
FeN_4	-653.34816337	-663.63744123	-659.55725226	-668.36721095
Fe,Mn/N_6-1	-642.28483595	-652.22323442	-647.83087899	-657.10386515
Fe,Mn/N_6-2	-652.22864543	-662.5056048	-658.03911335	-666.43719503
Fe,Mn/N_8-a (Fe site)	-878.95803849	-889.78169965	-885.20099546	-894.2525772
Fe,Mn/N_8-b (Mn site)		-889.81317513	-885.45183983	-894.15376072

Supplementary Table 12. The calculated free energy (eV) for the 4-elctron transfer processes during ORR at different reaciton corrdinations. The applied potential U=0 V.

Models	O_2+2H_2O	OH^-+H_2O+*OOH	2OH^-+*O+H_2O	3OH^-+OH*	4OH^-	onset potental (V)
MnN_4	4.92	3.373	1.194	0.422	0	0.422
FeN_4	4.92	3.445	1.174	0.811	0	0.364
Fe,Mn/N_6-1	4.92	3.668	1.873	1.154	0	0.719
Fe,Mn/N_6-2	4.92	4.277	1.611	0.776	0	0.644
Fe,Mn/N_8-a (Fe site)	4.92	3.169	1.141	0.276	0	0.276
Fe,Mn/N_8-b (Mn site)	4.92	3.289	0.927	0.198	0	0.198

Supplementary Table 13. The limiting overpotential for the structural models.

Model	MnN4	FeN4	Fe,Mn/N6 -1	Fe,Mn/N6 -2	Fe,Mn/N8 (Fe)	Fe,Mn/N8 (Mn)
Overpotential (eV)	0.81	0.87	0.51	0.59	0.95	1.03

Supplementary Table 14. The calculated vibrational frequencies of O, OH and OOH adsorbed on the support. The corresponding energy of ZPE-TS (ZPE: zero-point energy, T: temperture (300K), S: entropy) are also listed.

	Frequencies (meV)	ZPE-TS (eV)
O*	27.96, 34.74, 78.77	0.021
OH*	10.98, 23.47, 52.21, 53.82, 102.94, 457.36	0.248
OOH*	8.69, 15.18, 24.64, 24.82, 34.67, 60.88, 83.22, 151.69, 454.15	0.245

Supplementary Table 15. The calculated free energy (eV) for the 4$^-$elctron transfer processes during OER at different reaciton corrdinations.

Models	$4OH^-$	$3OH^-$+OH*	$2OH^-$+*O+H_2O	OH^-+H_2O+*OOH	O_2+$2H_2O$	onset potental (V)
MnN_4	0	1.7576	3.1646	3.1646	3.7968	2.1792
FeN_4	0	1.4595	3.3661	3.3661	4.1608	2.2702
Fe,Mn/N_6-1	0	0.6412	1.717	1.717	2.26	1.795
Fe,Mn/N_6-2	0	1.8895	3.7206	3.7206	5.7428	2.6657
Fe,Mn/N_8-a (Fe site)	0	1.7522	2.917	2.917	3.194	2.0285
Fe,Mn/N_8-b (Mn site)	0	2.1639	3.797	3.797	4.528	2.362

References

1 Li, J. *et al.* Atomically dispersed manganese catalysts for oxygen reduction in proton-exchange membrane fuel cells. *Nat. Catal.* **1**, 935-945 (2018).

2 Jiao, L. *et al.* From Metal-Organic Frameworks to Single-Atom Fe Implanted N-doped Porous Carbons: Efficient Oxygen Reduction in Both Alkaline and Acidic Media. *Angew. Chem. Int. Ed.* **57**, 8525-8529 (2018).

3 Strickland, K. *et al.* Highly active oxygen reduction non-platinum group metal electrocatalyst without direct metal-nitrogen coordination. *Nat. Commun.* **6**, 7343 (2015).

4 Ye, W. *et al.* Precisely tuning the number of Fe atoms in clusters on N-doped carbon toward acidic oxygen reduction reaction. *Cell Press: Chem.* **5**, 2865-2878 (2019).

5 Wang, Z. *et al.* Fe, Cu - Coordinated ZIF - Derived Carbon Framework for Efficient Oxygen Reduction Reaction and Zinc–Air Batteries. *Adv. Funct. Mater.* **28**, 1802596 (2018).

6 Hou, C. C. *et al.* Single-Atom Iron Catalysts on Overhang-Eave Carbon Cages for High-Performance Oxygen Reduction Reaction. *Angew. Chem. Int. Ed.* **59**, 7384-7389 (2020).

7 Li, J. *et al.* Ultrahigh-Loading Zinc Single-Atom Catalyst for Highly Efficient Oxygen Reduction in Both Acidic and Alkaline Media. *Angew. Chem. Int. Ed.* **58**, 7035-7039 (2019).

8 Xiao, F. *et al.* Nitrogen-coordinated single iron atom catalysts derived from metal organic frameworks for oxygen reduction reaction. *Nano Energy* **61**, 60-68 (2019).

9 Yang, Z. *et al.* Boosting Oxygen Reduction Catalysis with Fe–N_4 Sites Decorated Porous Carbons toward Fuel Cells. *ACS Catal.* **9**, 2158-2163 (2019).

10 Shinde, S. S. *et al.* Unveiling dual-linkage 3D hexaiminobenzene metal–organic frameworks towards long-lasting advanced reversible Zn–air batteries. *Energy Environ. Sci.* **12**, 727-738 (2019).

11 Pan, Y. *et al.* A Bimetallic Zn/Fe Polyphthalocyanine-Derived Single-Atom Fe-N_4 Catalytic Site:A Superior Trifunctional Catalyst for Overall Water Splitting and Zn-Air Batteries. *Angew. Chem. Int. Ed.* **57**, 8614-8618 (2018).

12 Yang, Y. *et al.* O-, N-Atoms-Coordinated Mn Cofactors within a Graphene Framework as Bioinspired Oxygen Reduction Reaction Electrocatalysts. *Adv. Mater.* **30**, e1801732 (2018).

13 Lin, Y. *et al.* Fabricating Single-Atom Catalysts from Chelating Metal in Open Frameworks. *Adv. Mater.* **31**, e1808193 (2019).

14 Chen, Y. *et al.* Isolated Single Iron Atoms Anchored on N-Doped Porous Carbon as an Efficient Electrocatalyst for the Oxygen Reduction Reaction. *Angew. Chem. Int. Ed.* **56**, 6937-6941 (2017).

15 Cheng, W. *et al.* Boosting defective carbon by anchoring well-defined atomically dispersed metal-N4 sites for ORR, OER, and Zn-air batteries. *Appl. Catal., B* **260,** 118198 (2020).

16 Chen, G. *et al.* Zinc-Mediated Template Synthesis of Fe-N-C Electrocatalysts with Densely Accessible Fe-Nx Active Sites for Efficient Oxygen Reduction. *Adv. Mater.* **32**, e1907399 (2020).

17 Zhang, X. *et al.* A General Method for Transition Metal Single Atoms Anchored on Honeycomb-Like Nitrogen-Doped Carbon Nanosheets. *Adv. Mater.* **32**, e1906905 (2020).
